# The conformational change of the protease inhibitor α_2_-macroglobulin is triggered by the retraction of the cleaved bait region from a central channel

**DOI:** 10.1016/j.jbc.2022.102230

**Published:** 2022-07-01

**Authors:** Seandean Lykke Harwood, Khang Diep, Nadia Sukusu Nielsen, Kathrine Tejlgård Jensen, Jan J. Enghild

**Affiliations:** Department of Molecular Biology and Genetics, Aarhus University, Aarhus, Denmark

**Keywords:** α_2_-macroglobulin, thiol ester, protease, proteinase, proteolysis, protease inhibition, inhibition mechanism, mutagenesis *in vitro*, protein engineering, α′NT, N-terminal region of the truncated α-chain, A2M, α2-macroglobulin, A2MF, α_2_-macroglobulin superfamily, A2ML1, A2M-like protein 1, A2Moo, *Xenopus laevis* ovomacroglobulin, ANA, anaphylactic domain, EXT, extended, HBS, Hepes-buffered saline, LNK, linker region, LRP1, low-density lipoprotein receptor–related protein 1, MG, macroglobulin domain, MMP, matrix metalloproteinase, nEXT, nonmodified extended, PRM, parallel reaction monitoring, TE, thiol ester domain

## Abstract

The protease inhibitor α_2_-macroglobulin (A2M) is a member of the ancient α_2_-macroglobulin superfamily (A2MF), which also includes structurally related proteins, such as complement factor C3. A2M and other A2MF proteins undergo an extensive conformational change upon cleavage of their bait region by proteases. However, the mechanism whereby cleavage triggers the change has not yet been determined. We have previously shown that A2M remains functional after completely replacing its bait region with glycine and serine residues. Here, we use this *tabula rasa* bait region to investigate several hypotheses for the triggering mechanism. When *tabula rasa* bait regions containing disulfide loops were elongated by reducing the disulfides, we found that A2M remained in its native conformation. In addition, cleavage within a disulfide loop did not trigger the conformational change until after the disulfide was reduced, indicating that the introduction of discontinuity into the bait region is essential to the trigger. Previously, A2MF structures have shown that the C-terminal end of the bait region (a.k.a. the N-terminal region of the truncated α chain) threads through a central channel in native A2MF proteins. Bait region cleavage abolishes this plug-in-channel arrangement, as the bait region retracts from the channel and the channel itself collapses. We found that mutagenesis of conserved plug-in-channel residues disrupted the formation of native A2M. These results provide experimental evidence for a structural hypothesis in which retraction of the bait region from this channel following cleavage and the channel’s subsequent collapse triggers the conformational change of A2M and other A2MF proteins.

Human α_2_-macroglobulin (A2M) is an abundant plasma protein (1–6 g/l) with multiple functions, including protease inhibition, chaperone-like activity, and cytokine binding (1, 2, 3, 4). It is the most thoroughly characterized protease inhibitor in the α_2_-macroglobulin superfamily (A2MF) of proteins, which also includes other protease inhibitors, such as pregnancy zone protein (PZP) and A2M-like protein 1 (A2ML1), complement factors such as C3, C4, and C5, and the transforming growth factor β coreceptor CD109. A shared characteristic of A2MF proteins is their ability to undergo a major conformational change induced when proteases cleave a designated susceptible region, that is, the bait region of A2MF protease inhibitors or the anaphylactic (ANA) domain of A2MF complement factors. A second shared A2MF characteristic is the formation of an internal thiol ester (TE) group in almost all superfamily members (5, 6, 7); this TE is protected in the original native conformation of A2MF proteins but becomes exposed during the proteolytically induced conformational change (8). In A2MF protease inhibitors, the TE is used to covalently conjugate the instigating protease (9), which becomes trapped inside the A2MF and is pseudoinhibited because of its sequestration from substrate proteins (2). In A2MF complement factors C3 and C4, a complement-activating surface (*e.g.*, a cell membrane or bacterial cell wall) is conjugated, anchoring the complement factor and locally amplifying the complement cascade through the formation of additional convertase complexes (10).

Crystal structures of both native C3 and protease-cleaved C3b were determined in the early 2000s, and the other complement factors followed shortly afterward (11, 12, 13, 14, 15, 16). More recently, high-resolution structures of native A2MF protease inhibitors such as bacterial protease inhibitors (17, 18), human A2ML1 (19), and frog (*Xenopus laevis*) ovomacroglobulin (A2Moo) (20) have been determined. These structures reveal that all A2MF proteins consist of ten core domains (eight macroglobulin [MG] domains, a “complement subcomponent C1r/C1s, urchin embryonic growth factor, and bone morphogenetic protein 1” [CUB] domain, and a TE domain, a linker [LNK] region, and the cleavable moiety [either the bait region or the ANA domain]) (Fig. 1*A*). They also confirm that the native conformations and the structural rearrangements upon cleavage are largely similar in A2MF protease inhibitors and complement factors, demonstrating that the conformational change is a conserved feature of A2MF proteins (Fig. 1*B*). A2M consists of four of these A2MF base units arranged in a homotetramer (Fig. 1*C*), and there is evidence that a typical A2MF proteolysis-induced conformational change is undergone by each subunit (21).

**Figure 1:**
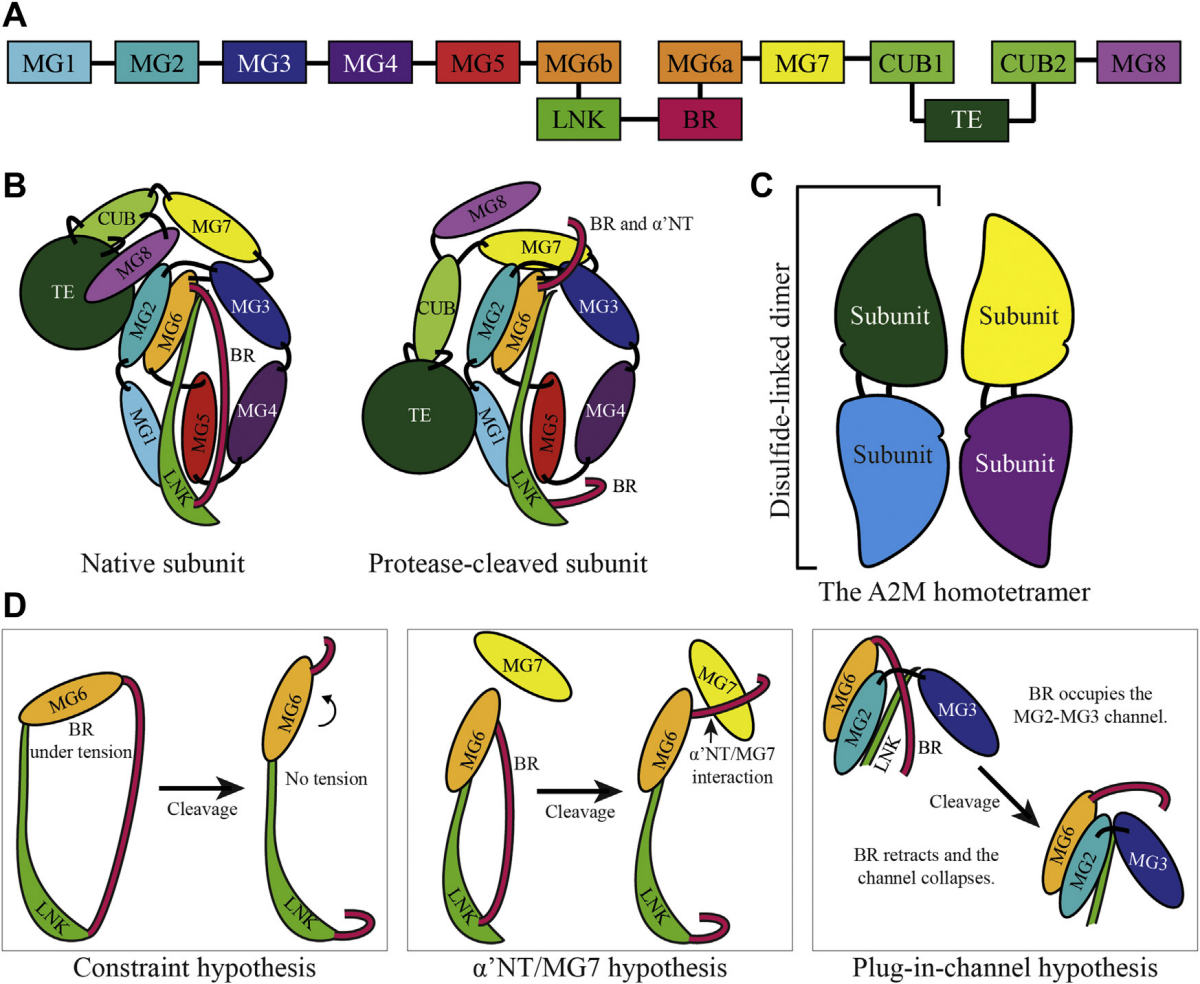
**Schematic representations of the structure of A2M and trigger hypotheses.***A*, the domain organization of A2M and other A2MF proteins, with eight macroglobulin (MG) domains, linker (LNK), and bait (BR) regions, a CUB domain, and the thiol ester (TE) domain. Note that the MG6 and CUB domains are interrupted by the inserted LNK and BR, and TE domain, respectively. *B*, a schematic representation of an A2M subunit in its native and protease-cleaved conformations. The same coloring as in *A* is used. These representations are based on structures of other A2MF proteins such as native C3 (PDB ID: 2A73) and A2ML1 (PDB ID: 7Q5Z), as well as methylamine-treated A2M (PDB ID: 4ACQ). In all structurally determined A2MF proteins, the major conformational changes following BR cleavage involve the MG7, CUB, TE, and MG8 domains, whereas the MG1–6 domains and LNK region provide a mostly static framework. *C*, the organization of A2M subunits into disulfide-linked dimers and the noncovalent dimer–dimer interactions producing the homotetramer. *D*, illustrations of the three triggering hypotheses investigated in this study. Only the domains relevant to each hypothesis are shown for simplicity. In the constraint hypothesis, the BR is originally under tension, and the connecting LNK and MG6 domains are held in constrained positions. When this tension is released by BR cleavage, the domains are free to move to more favorable positions. The conformational change of A2M could putatively proceed when the movement of MG6 disrupts the proximal TE domain. In the α′NT–MG7 hypothesis, the interactions between the α′NT region and MG7 domain that are seen in A2MF proteins after BR cleavage drive the conformational change, perhaps because of reorientation of the MG7 domain. In the plug-in-channel hypothesis, the occupation of the MG2–MG3 channel (which is also formed by the MG2–MG3 linker and the first α-helix of the LNK region) by the BR and α′NT region is required for the stability of the native conformation. When the BR is cleaved, the α′NT region retracts through the channel, which then collapses. The conformation change of A2M could putatively be driven by repositioning of the MG2 or MG3 domains, which are proximal to the TE and MG8 domains, respectively. α′NT, N-terminal region of the truncated α-chain; A2M, α_2_-macroglobulin; A2MF, α_2_-macroglobulin superfamily; A2ML1, A2M-like protein 1; PDB, Protein Data Bank.

Both the A2ML1 and A2Moo studies independently noted the threading of the C-terminal region to the bait region (referred to from here on by its name in C3, the N-terminal region of the truncated α-chain [α′NT]) through a hydrophobic channel formed by the MG2, MG3, and LNK domains. This arrangement is conserved in all currently determined native A2MF structures and is disrupted after proteolytic cleavage. Cleavage allows the α′NT region to retract through the channel to the other side of the A2MF protein, where it interacts with the MG7 domain, and the channel collapses. In activated complement factors C3b and C4b, the interacting α′NT–MG7 serve as a binding site for factor B and factor C2, respectively (22, 23). Based on these structures, the plug-in-channel hypothesis has been proposed to explain the molecular trigger for the A2MF conformational change, through displacement of the MG2–MG6 domains during the channel’s collapse and subsequent disruption of the hydrophobic TE–MG8 interface (20).

A2M and some other A2MFs such as C3 also undergo a conformational change following direct aminolysis of the TE using small nucleophiles such as methylamine. These react with the TE even while it is protected in the native conformation (24, 25). The resulting aminolyzed conformation is structurally similar to the protease-cleaved conformation. However, proteolysis induces a rapid conformational change (<15 s (26)), whereas aminolysis induces a slower conformational change that takes minutes (27). Chemical modification of the aminolyzed TE with hydrophobic groups can stabilize the native conformation (28), indicating that the aminolysis-induced conformational change occurs because of the generation of polar groups in the previously hydrophobic TE environment and is likely to be mechanistically distinct from the proteolysis-induced conformational change. TE aminolysis or hydrolysis is not necessary for the conformational change, as demonstrated by A2MF proteins that lack TEs (such as complement C5) as well as A2M and C3 with their TEs replaced with disulfides by mutation (29).

We have previously shown that a recombinant A2M molecule where all 39 bait region residues (P690–T728) were replaced by 13 GGS triplets was mostly produced as native A2M, and that these *tabula rasa* A2Ms were fully functional as protease inhibitors if cleavage sites were reintroduced into the bait region (30). These results demonstrated that no part of the bait region sequence is essential to the native conformation or the conformational change of A2M. In this study, we use the *tabula rasa* bait region as the basis for further bait region engineering and investigate several possible trigger hypotheses to explain the triggering of the conformational change of A2M after bait region cleavage (Fig. 1*D*). We show that extension of the bait region without proteolysis (by reduction of disulfide loops incorporated into the *tabula rasa* bait region) or cleavage within a disulfide loop cannot trigger the conformational change of A2M. This indicates that the connectivity of the bait region is critical to maintaining the native conformation of A2M, as predicted by the plug-in-channel hypothesis. We proceeded to mutate key residues in the α′NT region and its channel, which perturbed the formation of native A2M. Altogether, these results provide experimental support for the plug-in-channel hypothesis as the molecular trigger for the A2MF conformational change.

## Results

### Extension of the bait region sequence predisposes A2M toward its fully collapsed conformation

In our previous study of *tabula rasa* A2M with an engineered bait region consisting entirely of glycine and serine residues, we noted that *tabula rasa* A2M with a 39-residue bait region (equivalent to the wildtype bait region) was produced not only in a native conformation (with a slow electrophoretic mobility in pore-limited native PAGE) but with a significant content of both fast- and intermediate-migrating A2M (probably corresponding to fully and partially collapsed A2M tetramers, respectively), whereas wildtype A2M was almost exclusively native (30). If the wildtype bait region has secondary structure or participates in any interactions, then it is effectively shorter than a *tabula rasa* bait region of equivalent length, and we speculated that the effectively longer bait region of *tabula rasa* A2Ms might underly their increased content of collapsed A2M. Indeed, shortening the *tabula rasa* bait region by 7 to 32 residues restored its native content to that of the wildtype. These results suggested that the length of the bait region is important to the formation or stability of the native conformation of A2M.

**Figure 2:**
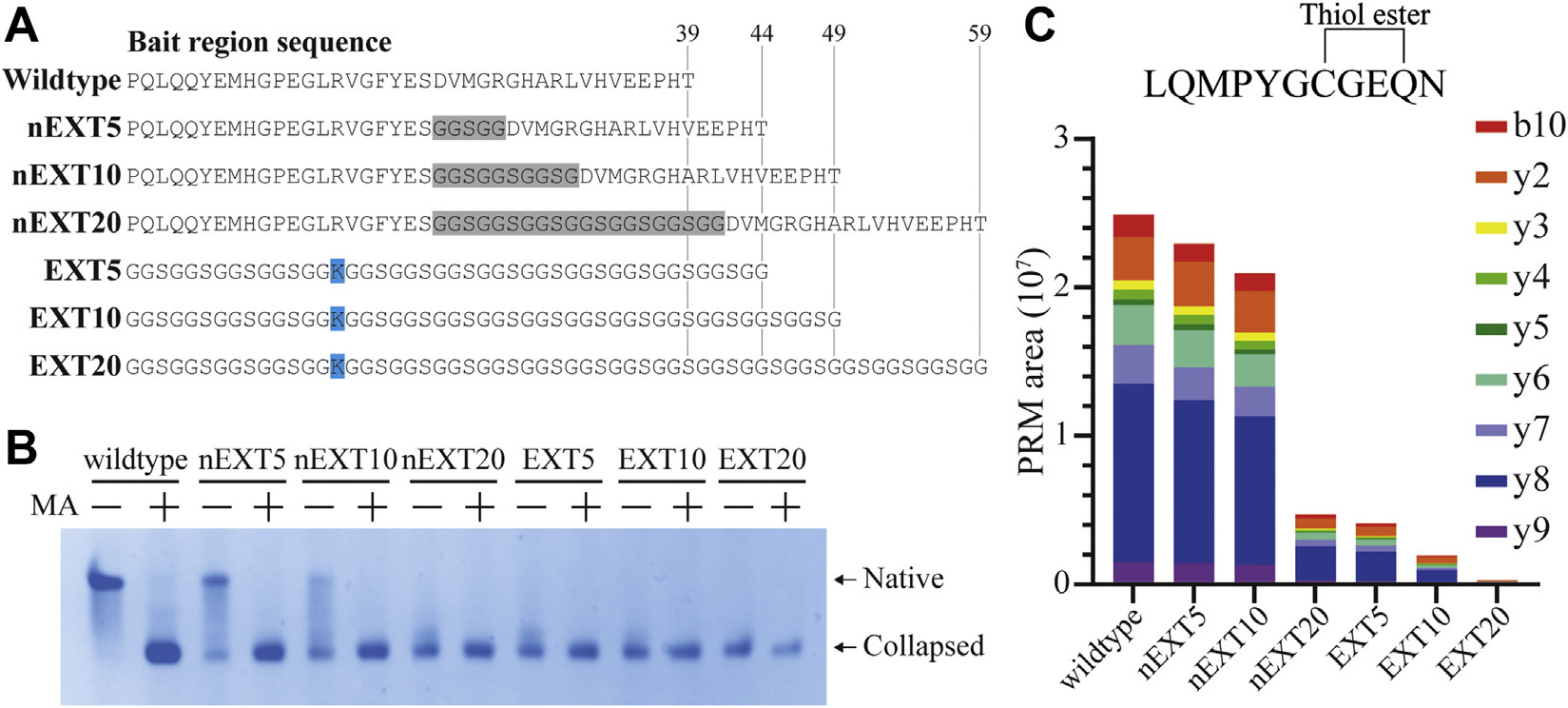
**Investigating bait region (BR) length as a factor in the formation of native A2M.***A*, sequences for the nEXT and EXT BRs, which increase the length of the wildtype or TR K704 BR, respectively, through the addition of glycine and serine residues. Length increases of 5, 10, and 20 residues were tested. *B*, pore-limited native PAGE of conditioned media containing each of these seven A2Ms; where indicated, the samples were treated with methylamine (MA) prior to electrophoresis. Extending the BR sequence caused A2M to be expressed in its collapsed conformation to a greater extent. A2M with *tabula rasa*–based BRs became predisposed to the collapsed conformation after extension by five residues, in contrast to the wildtype BR, which required extension between 10 and 20 residues before it was predominantly collapsed. *C*, all tetrameric A2M from untreated A2M samples separated by pore-limited native PAGE was digested in-gel by pepsin and analyzed by LC–MS/MS. The thiol ester abundance was quantified using PRM of the indicated MS2 product ions. Thiol ester abundance correlated well to the native A2M content determined in *B*. A2M, α_2_-macroglobulin; EXT, extended; nEXT, nonmodified extended; PRM, parallel reaction monitoring.

We hypothesized that extending the length of the bait region might decrease the stability of native A2M because the bait region functions as a constraint upon the domains at its N- and C-terminal ends, like a bowstring that is pulled taut. Bait region cleavage would then trigger the conformational change of A2M by removing this constraint, releasing the metaphorical bowstring, and allowing the “bow” to transition into a preferred conformation (Fig. 1*D*). According to this constraint hypothesis, a sufficiently extended bait region can no longer constrain the native conformation of A2M and will trigger the conformational change of A2M similarly to bait region cleavage. To investigate, we expressed six new A2Ms in which the sequence of the wildtype or *tabula rasa* bait region was extended by 5, 10, or 20 residues, designated as nonmodified extended (nEXT) or extended (EXT) A2M, respectively (Fig. 2*A*). Pore-limited native PAGE showed that this lengthening of the bait region predisposed the resulting A2M toward a collapsed conformation; the majority of *tabula rasa* A2M was collapsed after extension by only five residues, whereas the critical length for wildtype A2M appeared to lie between extension by 10 to 20 residues (Fig. 2*B*). This is consistent with an apparent difference of approximately seven residues in effective length between the wildtype and *tabula rasa* bait regions. The TE abundance in these A2M proteins was determined by LC–MS/MS with parallel reaction monitoring (PRM) of the LQMPYGCGEQN peptide covering the TE; the results showed that TE abundance correlated to the amount of native A2M as determined by pore-limited native PAGE (Fig. 2*C*). Reducing SDS-PAGE showed that the A2M EXT proteins were not bait region cleaved (Fig. S1), and therefore, their predisposition toward a collapsed conformation did not result from an increased susceptibility to cleavage by cell culture proteases. These results show that increasing the length of the bait region lowers the content of native A2M in recombinant A2M. However, the EXT and nEXT mutations lengthen the bait region during translation, before A2M assumes its native conformation. These results do not determine whether the bait region lengthening destabilized the native conformation (as predicted by the constraint hypothesis) or prevented A2M from assuming its native conformation in the first place (in which case, the results are irrelevant for the mechanism by which native A2M is triggered to change its conformation). As we will proceed to show, the latter explanation is most likely.

### Bait region cleavage or extension without disrupting its continuity does not cause A2M to collapse

**Figure 3:**
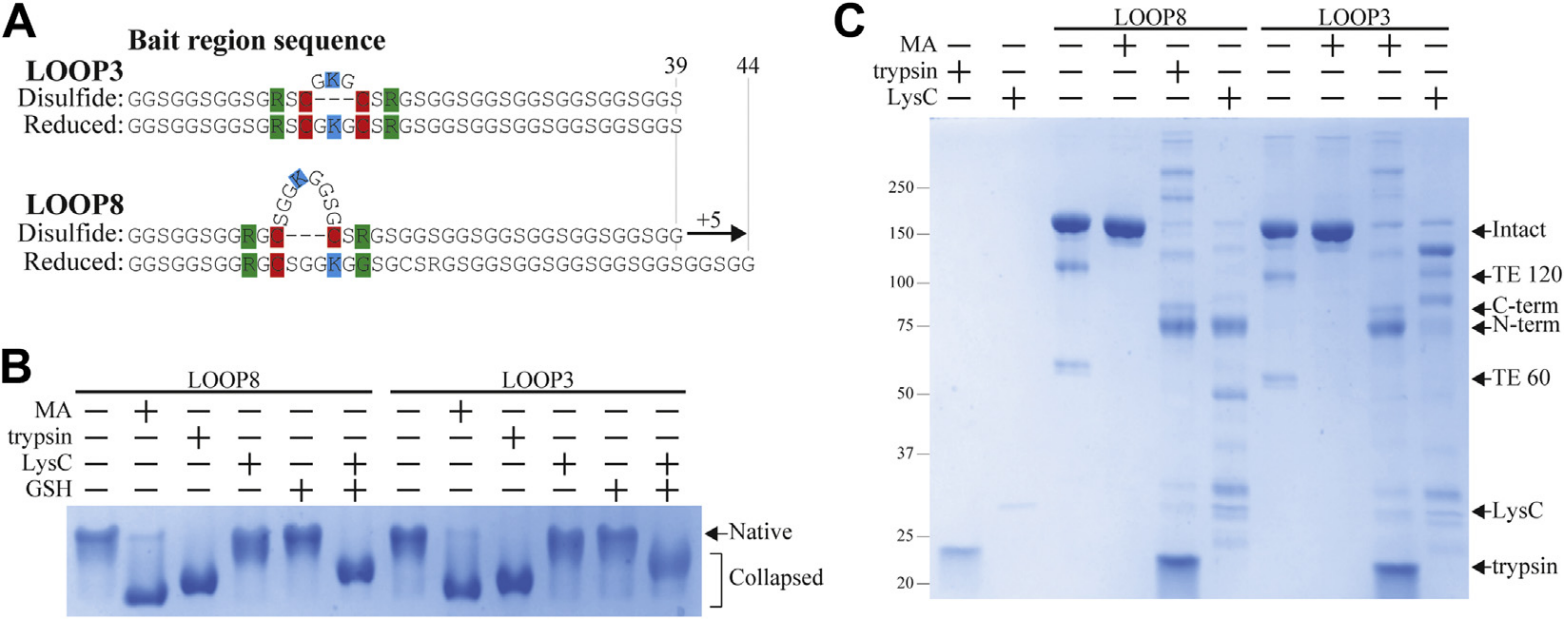
**Conformational changes of A2M LOOP8 and LOOP3.***A*, the bait region (BR) sequences for *tabula rasa* BRs with disulfide-linked hairpin loops are given. LOOP3 has three residues within its disulfide-linked loop, whereas LOOP8 has eight residues. Both LOOP constructs have lysine residues in their loops, allowing loop formation to be assessed using LysC, as well as an arginine residue outside the loop, which can be cleaved by trypsin. *B*, pore-limited native PAGE of purified A2M with the LOOP3 and LOOP8 BRs (where the native content of A2M LOOP8 was enriched by LRP1 depletion), treated as indicated with methylamine (MA), trypsin, or LysC. Where indicated, control or LysC-cleaved A2M samples were reduced using 1 mM GSH. Both A2Ms were collapsed by MA or trypsin. Neither was initially collapsed by LysC cleavage, but both A2Ms collapsed upon reduction of its BR disulfides after LysC cleavage. Reduction with GSH without prior LysC cleavage did not cause a conformational change, indicating that an extension of the length of BR by five residues does not approximate the conformational change’s trigger. *C*, reducing SDS-PAGE showing MA treatment and proteolysis of both A2Ms. The autolytic bands formed from fragmentation at the thiol ester (TE) site when it is heated under denaturing conditions are labeled TE 120 and TE 60, whereas the fragments arising from proteolytic BR cleavage are labeled C-term and N-term. TE activation is decoupled from BR cleavage in both LOOP proteins when they are cleaved by LysC, as no high–molecular weight conjugation products are formed. This indicates that BR cleavage by LysC, inside the disulfide-bridged loop, does not trigger conformational change. A2M, α_2_-macroglobulin.

Our experiment with the EXT and nEXT A2M proteins did not determine whether this extension of the bait region during protein translation decreased the stability of native A2M in accordance with the constraint hypothesis or instead impaired initial assumption of A2M of its native conformation, which necessarily happens after bait region translation. To distinguish these two possibilities, we designed new *tabula rasa* A2Ms with bait regions that could be extended without proteolysis after A2M had already assumed its native conformation. This was accomplished by incorporating disulfide-linked hairpin loops into the *tabula rasa* bait region, so that disulfide formation would exclude the intermittent residues from contributing to the effective length of the bait region (Fig. 3*A*). The selective reduction of these hairpin loops would then extend the bait region by the difference in length between the disulfide-excluded residues and the disulfide bond (which has an inter-Cα length approximately equivalent to that of three residues). These constructs included lysine residues within the hairpin loops that can be selectively cleaved by the lysine-specific protease LysC and arginine residues outside the loops that can be cleaved by trypsin, which is specific to both arginine and lysine residues.

Initially, two *tabula rasa* bait regions with disulfide-linked loops were tested: LOOP3 and LOOP8, which had three and eight residues in the loops between their cysteines, respectively (Fig. 3*A*). LOOP3 was designed as a control that would not extend upon reduction of its disulfide. In contrast, LOOP8 would extend by five residues to the same bait region length as A2M EXT5, which was produced almost completely in a collapsed conformation (Fig. 2). After expression and purification, LOOP8 was approximately 50% native, indicating that the bait region disulfides formed sporadically but that when they formed, they effectively shortened the bait region and enabled the formation of native A2M (Fig. S2). To purify LOOP8 in the native A2M conformation, collapsed LOOP8 was depleted using a low-density lipoprotein receptor–related protein 1 (LRP1) resin (Fig. S2). The native conformation of LOOP3 and LRP1-depleted LOOP8 was demonstrated by their migration in pore-limited native PAGE (Fig. 3*B*) and the presence of TE-dependent fragments in SDS-PAGE (formed when the TE is heated in denaturing conditions during sample preparation) (Fig. 3*C*). Both LOOP8 and LOOP3 underwent a conformational change upon TE aminolysis by methylamine or bait region cleavage by trypsin (Fig. 3*B*). However, neither LOOP changed conformation after cleavage by LysC, despite bait region cleavage occurring as determined by reducing SDS-PAGE (Fig. 3, *B* and *C*), demonstrating that bait region cleavage is insufficient to trigger the conformational change of A2M if the bait region remains contiguous after cleavage.

To investigate whether reduction of the bait region disulfides would trigger the conformational change of A2M by extending the bait region, we required a way to selectively reduce the LOOP disulfides without reducing endogenous intersubunit disulfides of A2M. We hypothesized that relatively large reducing agents such as GSH (307 Da) or thioredoxin (12 kDa) might preferentially reduce the exposed bait region disulfides, and a titration experiment identified conditions (*e.g.*, 1 mM GSH) that reduced the LOOP3 disulfide without dissociating the A2M tetramer (Fig. S3). Reduction of LysC-cleaved LOOP8 and LOOP3 caused them to change conformations (Fig. 3*B*), showing that a delayed cleavage of the bait region by reduction, long after the moment of proteolysis, can still trigger the conformational change of A2M. However, reduction of either LOOP8 or LOOP3 with GSH without LysC cleavage did not induce any conformational change (Fig. 3*B*), despite the extension of the LOOP8 bait region to 44 residues (equivalent to EXT5) upon reduction. These results indicate that the predisposition toward collapsed A2M seen when the bait region was lengthened in the nEXT and EXT mutants is not because of a destabilization of the native conformation when the bait region is lengthened, as predicted by the constraint hypothesis. Instead, it is likely that lengthening of the bait region during translation in nEXT and EXT mutants prevented A2M from assuming its native conformation in the first place.

**Figure 4:**
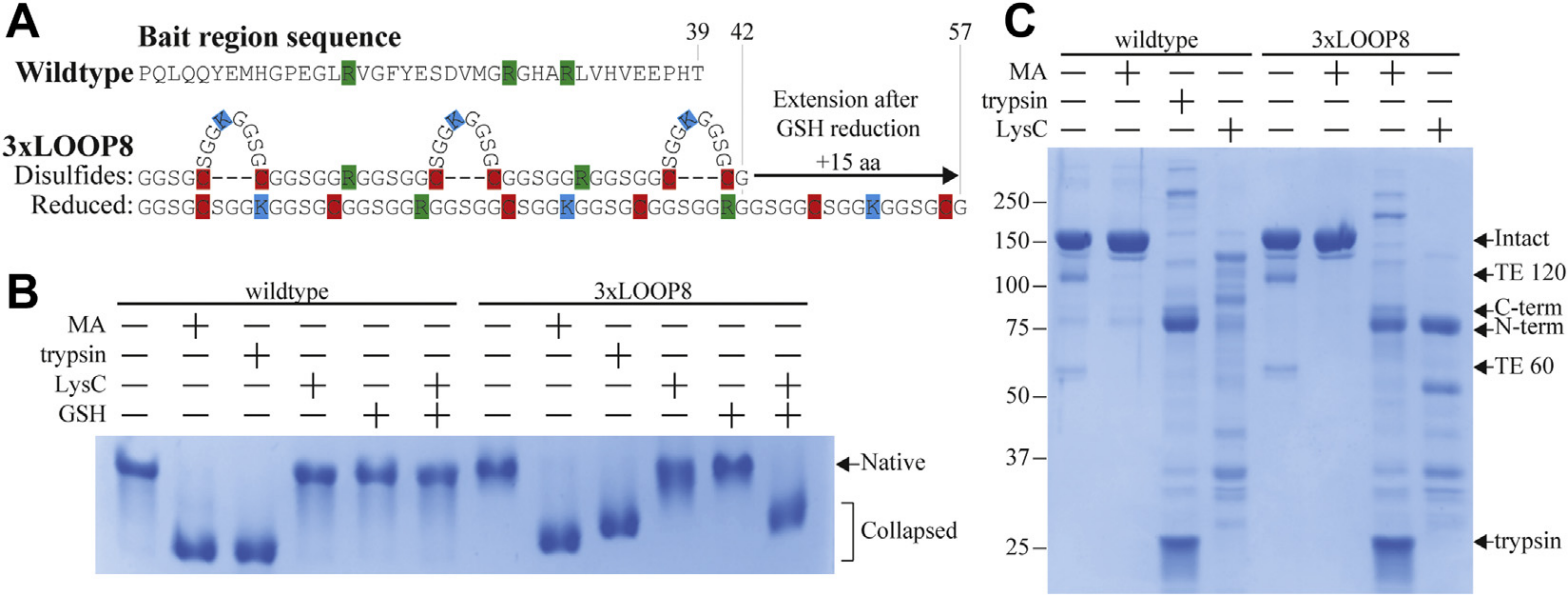
**Mechanistic study of the bait region (BR) of A2M using disulfide-linked hairpin loops.***A*, the BR sequence for a *tabula rasa* BR with three disulfide-linked hairpin loops is given. This sequence, 3xLOOP8, has three disulfides, which each exclude eight residues from contributing to the effective length of the BR. Each disulfide loop contains a lysine residue that is cleavable by LysC or trypsin. Between the loops, there are arginine residues that are only cleavable by trypsin. *B*, pore-limited native PAGE of wildtype A2M and A2M 3xLOOP8 (after LRP1 depletion), treated as indicated with methylamine (MA), trypsin, or LysC. Where indicated, control or LysC-cleaved A2M samples were in addition reducing using 1 mM GSH. Native A2M incorporating 3xLOOP8 was still produced despite the BR length of 57 residues, which is comparable to that of the entirely collapsed A2M EXT20 from Figure 2. Both A2Ms were collapsed by methylamine or trypsin. A2M 3xLOOP8 was not initially collapsed by LysC cleavage but collapsed upon reduction of its BR disulfides after LysC cleavage. A2M 3xLOOP8 did not collapse after reduction without prior proteolysis, showing that an extension of the BR length by 15 amino acids on the protein level does not trigger the conformational change of A2M. *C*, reducing SDS-PAGE showing methylamine treatment and proteolysis of both A2Ms. The autolytic bands formed from fragmentation at the thiol ester (TE) site when it is heated under denaturing conditions are labeled TE 120 and TE 60, whereas the fragments arising from proteolytic BR cleavage are labeled C-term and N-term. TE activation is decoupled from BR cleavage in A2M 3xLOOP8 when it is cleaved by LysC, as it fails to form the high–molecular weight multimer products seen upon trypsin cleavage. A2M, α_2_-macroglobulin; EXT, extended; LRP1, low-density lipoprotein receptor–related protein 1.

It remained possible that extension by more than five residues was required to release the putative constraint imposed by the bait region. We therefore designed a LOOP23 bait region with 23 residues in its disulfide hairpin loop, but this long distance between the bait region cysteines apparently precluded disulfide formation as no native A2M LOOP23 was expressed. Instead, we designed the 3xLOOP8 bait region, which has three 8-residue disulfide loops with 11 residues separating the loops to enforce the intended cysteine pairing and extends from 42 to 57 residues upon reduction (Fig. 4*A*). As with LOOP8, a significant amount of collapsed 3xLOOP8 was expressed, and native 3xLOOP8 was therefore enriched by LRP1 resin depletion prior to experiments (Fig. S2). A2M 3xLOOP8 functioned essentially identically to A2M with the LOOP8 and LOOP3 bait regions; cleavage by LysC only triggered a conformational change after GSH reduction, demonstrating that the disulfide loops were formed, but GSH reduction alone did not trigger a conformational change (Fig. 4, *B* and *C*). Therefore, extension of the bait region by 15 residues to a total length of 57 residues did not trigger a conformational change in A2M. A bait region length of 57 residues is equivalent to nearly 200 Å; considering that an A2M subunit measures approximately 110 Å on its longest axis, we consider it unlikely that the putative structural constraint imposed by the bait region would not be alleviated by this extension. Therefore, we conclude that the conformational change of A2M cannot be triggered by bait region extension, falsifying the constraint hypothesis.

### The α′NT region is threaded through a channel, and disruption of this arrangement disrupts native A2M formation

**Figure 5:**
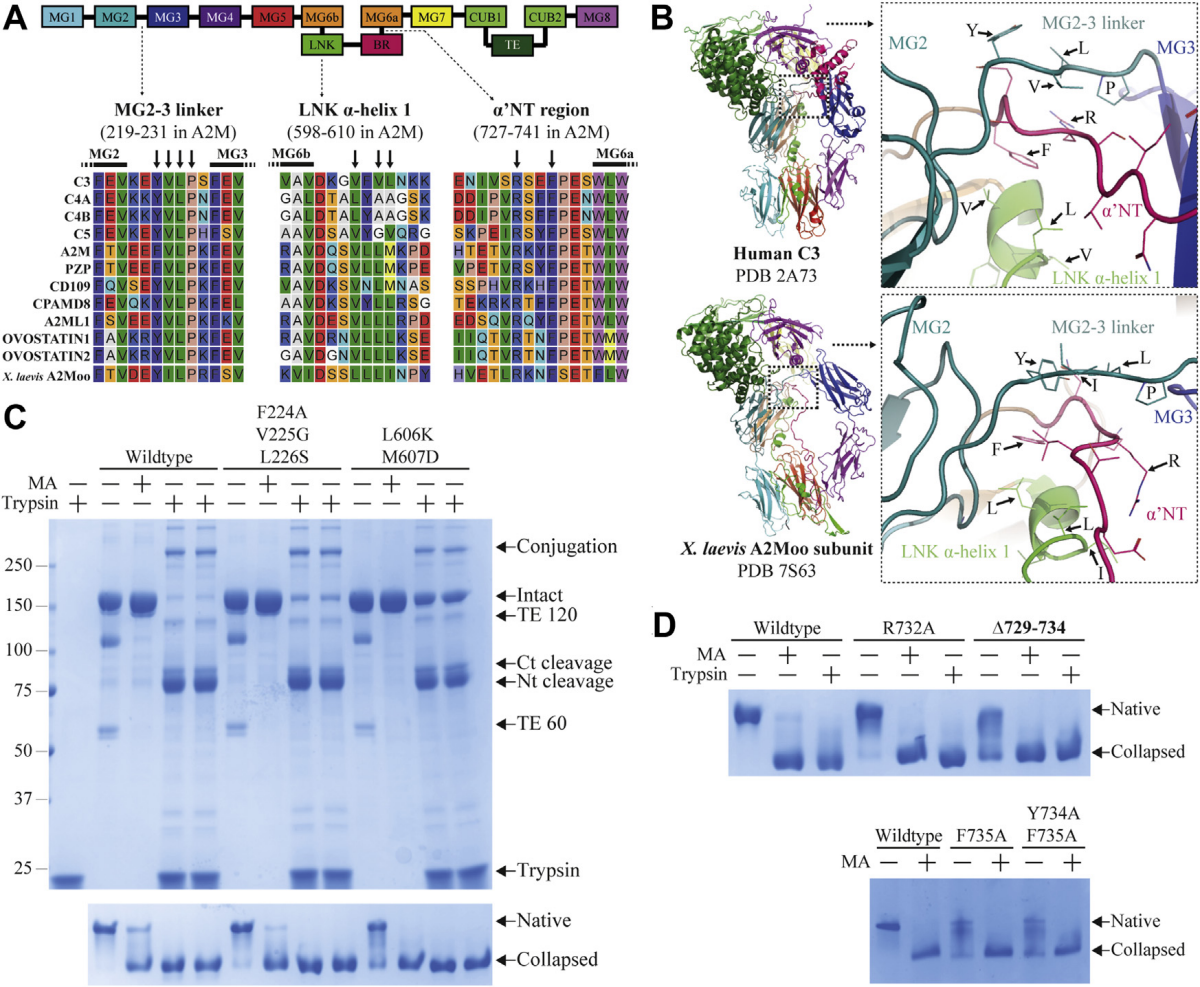
**Investigating the role of the α′NT region and channel.***A*, sequence alignment of all human A2MF proteins and *Xenopus laevis* ovomacroglobulin (A2Moo) in the vicinity of the MG2–MG3 linker, the first α-helix in the linker (LNK) region, and the α′NT region. Residues of suspected importance are indicated with *arrows*. *B*, structures of the α′NT channel in native C3 and one subunit of the *X. laevis* A2Moo tetramer. The indicated residues from *A* are also indicated here. The α′NT region threads through a channel comprised by the first LNK α-helix from below and the MG2–MG3 linker from above. *C*, recombinant A2M that was either wildtype, mutated in the MG2–MG3 linker by F224A–V225G–L226S, or in the LNK α-helix by L606K–M607D was treated with methylamine (MA) or trypsin (using two stocks of trypsin prepared at different times but otherwise identical), and analyzed by reducing SDS-PAGE or pore-limited native PAGE. The autolytic bands formed from fragmentation at the thiol ester (TE) site are labeled TE 120 and TE 60, whereas the fragments arising from proteolytic BR cleavage are labeled Ct cleavage and Nt cleavage. Both mutants undergo conformational change, but an increased amount of each mutant was produced in a collapsed conformation that migrated further than native A2M in pore-limited native PAGE and was not cleaved in its BR by a surplus of trypsin, as determined by SDS-PAGE. *D*, recombinant A2M with the R732A, Δ729 to 734 (replacement of these residues with GGSGGS), F735A, and combined Y734A–F735A mutations were compared with wildtype A2M in pore-limited native PAGE. All mutants had an increased content of collapsed A2M, although some native A2M was nonetheless produced that could still undergo conformational change. α′NT, N-terminal region of the truncated α-chain; A2M, α_2_-macroglobulin; A2MF, α_2_-macroglobulin superfamily.

Our results with the EXT and LOOP proteins showed that the bait region does not appear to constrain the native conformation of A2M through tension, necessitating a new hypothesis to explain the triggering mechanism of A2M. Several alternatives are already excluded by our data; the *tabula rasa* bait region itself revealed that the bait region does not participate in sequence-dependent interactions that either stabilize the native conformation or trigger the conformational change. Furthermore, the experiments with LOOP proteins show that bait region cleavage is not a trigger if the bait region is held together by a disulfide after cleavage and that triggering is possible with reduction instead of peptide backbone proteolysis. This shows that interactions with the instigating protease are neither sufficient nor necessary to initiate the triggering mechanism. To develop a new hypothesis, we turned to high-resolution structures of A2MF proteins such as C3 and a tetrameric ovomacroglobulin (A2Moo) from the African clawed frog (*Xenopus laevis*) (20), as there is currently no high-resolution structure for native human A2M. Complement factors have a structured ANA domain instead of an unstructured bait region; importantly, the ANA domain is intracellularly processed by furin at its N terminus (31), and this lack of connectivity between the LNK region and the ANA domain suggests that the triggering mechanism must propagate in the C-terminal direction in complement factors and, assuming a conserved mechanism, all A2MF proteins. The α′NT region lies between the ANA domain and the MG6a domain and, in contrast to the ANA and bait region, appears to be partially conserved among A2MF proteins, especially R732 and F735 (by the numbering of A2M) (Fig. 5*A*). In structures of native A2MF proteins, the α′NT region is threaded through a highly conserved channel surrounded by the MG2 and MG3 domains (to the sides), the MG2–MG3 LNK (from above), and the first α-helix in the LNK region (from below) (Fig. 5*B*) (11, 20). In the structures of A2MF proteins that have undergone their conformational change, the channel is collapsed and the α′NT region has been pulled through the channel to the other side of the A2MF protein where it interacts with the MG7 domain through a conserved ionic interaction between R732 and an acidic residue on the MG7 domain (D897 in A2M) (Fig. S4, *A* and *B*) (12, 32).

We formed two hypotheses based on these structural observations. In the plug-in-channel hypothesis, the occupation of the channel by the α′NT region stabilizes the native A2MF conformation, and cleavage of the bait region or ANA domain releases the α′NT region, thus triggering the conformational change (Fig. 1*D*). In the α′NT–MG7 hypothesis, it is the interaction between the α′NT region and the MG7 domain after cleavage, which drives the conformational change (Fig. 1*D*). To test these hypotheses, we expressed several A2M proteins with mutations in the α′NT region and the surrounding channel. The combined F224A–V225G–L226S mutations replaced a highly conserved hydrophobic stretch of the MG2–MG3 LNK with small hydrophilic residues, whereas the L606K–M607D mutations replace small (in the case of C4 and C5) or hydrophobic (in the case of all other human A2MFs) residues in the LNK α-helix that oriented toward the α′NT region with large hydrophilic residues. Both sets of mutations, especially L606K–M607D, predisposed A2M toward a collapsed conformation as assessed by (i) reducing SDS-PAGE (where collapsed A2M was not cleavable even by a surplus of trypsin because of inaccessibility of the bait region) and (ii) pore-limited native PAGE, where the conformation of A2M is more directly observed; the remaining native A2M content underwent an apparently normal methylamine- and trypsin-induced conformational change (Fig. 5*C*). In the α′NT region, the R732A mutation, combined E729G–T730G–V731S–R732G–K733G–Y734S mutations (referred to as Δ729–734), F735A, and combined Y734A–F735A mutations were tested. R732A slightly increased the amount of collapsed A2M but did not affect the conformational change of A2M, whereas the other three mutations gave markedly increased amounts of collapsed A2M that were comparable to that of L606K–M607D (Fig. 5*D*). The ability of the R732A and Δ729 to 734 mutants to undergo normal proteolysis-induced conformational change indicates that these side chains do not participate in α′NT–MG7 interactions that are necessary for the trigger, falsifying the α′NT–MG7 hypothesis. On the other hand, the increase in collapsed A2M because of mutations in the α′NT region, especially F735A, and the surrounding channel indicated that disruption of the channel can prevent formation of native A2M, supporting the importance of the channel’s occupation for native A2MFs as predicted by the plug-in-channel hypothesis. In addition to the elimination of the constraint hypothesis and interaction-driven explanations, these results experimentally support the hypothesis that the A2MF conformational change is triggered by retraction of the α′NT region from the MG2–MG3–LNK channel following proteolysis.

## Discussion

This study investigated the triggering mechanism for the proteolysis-induced conformational change of A2M. Since the first formulation of the trap hypothesis for the protease-inhibitory mechanism of A2M, it has been of interest to elucidate how bait region cleavage triggers the conformational change. Initially, the tetrameric nature of A2Ms made it possible that the trigger operated across multiple subunits, but as the familial relations between A2M and other proteolytically activated TE proteins such as the monomeric complement factors became better understood, the existence of a conserved triggering mechanism in the A2MF proteins became increasingly likely. This rules out intersubunit interactions and furthermore requires that the mechanism be compatible both with the A2MF protease inhibitors’ bait region as well as the A2MF complement factors’ ANA domain. It has previously been proposed that the proteolytic removal of the ANA domain disrupts interactions between the ANA, MG3, and MG8 domains in αM complement factors, thus triggering their conformational change (14), and in fact, this seems like the obvious structural explanation for the triggering mechanism in A2MF complement factors. However, the bait region has no motifs that are essential to the conformational change, as indicated by the low conservation of the bait region sequence across species (33) and demonstrated by *tabula rasa* A2M (30). Therefore, perturbation of interactions between the cleavable moiety and the MG3–MG8 domains cannot be the A2MF trigger (assuming a conserved mechanism). Considering the high degree of preferential cleavage of the bait region or ANA domain by proteases, another hypothesis involves the existence of tension within the cleavable moiety that is released by its cleavage to trigger conformational change. Here, we investigated this hypothesis with the LOOP A2M proteins and found that an extension of the bait region by 15 residues did not trigger the conformational change of A2M, thereby ruling out the hypothesis of tension or constraint.

Crystal structures of both native C3 and protease-cleaved C3b were determined in the early 2000s, and the other complement factors followed shortly afterward (11, 12). More recently, high-resolution structures of native A2MF protease inhibitors, such as bacterial protease inhibitors (17, 18), human A2ML1 (19), and A2Moo (20), have been determined, further demonstrating that the conformational change is highly conserved in A2MF complement factors and protease inhibitors. Both the A2ML1 and A2Moo studies independently observed the threading of the α′NT region C-terminal to the bait region through a central channel in the native protease inhibitors as well as the fact that this arrangement is conserved in all native A2MF structures. They furthermore noted that this arrangement is disrupted after proteolytic cleavage, involving retraction of the bait region and α′NT region through the channel (to interact with the MG7 domain) and collapse of the channel and propose this as the triggering mechanism for the conformational change. Here, we have investigated this trigger hypothesis by mutating key residues in the α′NT region and the occupied channel. The R732A mutant underwent a normal protease-induced conformational change, indicating that the α′NT–MG7 interaction with an important R732–D897 ionic pairing happens after the trigger. Mutations in the α′NT region (especially of F735) and in the surrounding channel (especially in the LNK α-helix) increased the amount of collapsed A2M that was recombinantly produced, providing additional experimental support to the necessity of the plug-in-channel arrangement for the native A2MF conformation. Nonetheless, the hypothesis will benefit from additional experimental challenge, for example, by investigating whether native A2MF formation can be completely prevented by complete blocking of the channel (as only partial disruption of native A2MF formation by mutations around the plug-in-channel arrangement was achieved in this study), and whether disruption of the plug-in-channel arrangement by another method than proteolysis or reduction can trigger the A2MF conformational change.

While the *tabula rasa* bait region provides mechanistic insights into A2M, it was initially designed as the basis for engineering A2M proteins with increased specificity toward target proteases, for example, matrix metalloproteinases (MMPs) (30). The results of this study help to clarify design limitations in these engineered bait regions, as the EXT and nEXT results show that the length of the engineered bait regions affects the formation of native A2M. Furthermore, the LOOP results show that disulfides can be incorporated into the *tabula rasa* bait region, which makes new engineering opportunities available. For example, new sequences can be incorporated into disulfide loops, which, if cleaved, will not trigger the conformational change of A2M. More complex protease recognition sequences could then be incorporated into the bait region by distributing the sequence both inside and outside the disulfide loop. It is also possible that secondary structure (*e.g.*, paired β-strands or coiled-coil α-helix interactions) could be used to exclude loops instead of disulfides. Alternatively, A2M with cleavage sites entirely inside of LOOPs could be designed as redox-responsive protease inhibitors, for example, to restrict their protease inhibition to reducing environments such as tumors (34). In the future, the plug-in-channel hypothesis may even enable alternative triggers to proteolysis and reduction, such as interaction-based triggers, allowing A2MF proteins to function as logic gates in next-generation therapeutics.

A2MF proteins share a conserved conformational change, which likely arose in an ancestor protein in an early animal and through gene duplication has been turned to multiple functions, including (i) protease inhibition, (ii) the controlled amplification of the complement cascade, and (iii) other yet undiscovered functions, considering the existence of the A2MF transforming growth factor β coreceptor CD109 and other human proteins with currently unknown functions like CPAMD8 (C3 and PZP like alpha-2-macroglobulin domain containing 8) and the ovostatin homologs. Here, we have elaborated the mechanism by which bait region cleavage triggers the conformational change of A2M using new engineered bait regions. These results further substantiate the importance of the plug-in-channel arrangement, which mounting evidence indicates may constitute the molecular trigger for the A2MF conformational change.

## Experimental procedures

### Gene design

A pcDNA3.1(+) plasmid with the gene for wildtype A2M under control of a cytomegalovirus promoter was used for recombinant expression of A2M in a mammalian system (35). The bait region sequence was changed using either site-directed mutagenesis or synthesis of new bait region sequences followed by restriction site cloning, depending on the extent of the changes. This cloning work was performed by GenScript. All bait region sequences are shown in Table S1, and all full protein sequences are included in the supporting information.

### Expression and purification of A2M

All recombinant A2Ms were expressed in human embryonic kidney 293 FreeStyle cells using a standard transient transfection protocol. Briefly, 25 kDa linear polyethyleneimine (Polysciences) and plasmid DNA were incubated for 10 min in antibiotic-free FreeStyle medium (Thermo Fisher Scientific) at a 4:1 w/w polyethyleneimine:DNA ratio, then slowly dripped into a culture of cells at a density of one million cells per milliliter, to a final DNA concentration of 1 μg per milliliter culture. After 4 days, the supernatant was harvested by spinning down the cells at 1500*g* and adding Hepes (pH 7.4) to a final concentration of 50 mM.

Purification of recombinant A2M was performed using an established protocol with slight modifications (36, 37). Supernatants were first run through a Zn^2+^-loaded Chelating HiTrap column (GE Healthcare) and eluted with 50 mM EDTA, 150 mM NaCl, 100 mM sodium acetate, and pH 7.4. The EDTA eluate was dialyzed against 20 mM Hepes at pH 7.4, then loaded onto a HiTrap Q column (GE Healthcare), and eluted over a gradient of 0 to 400 mM NaCl (with a constant 20 mM Hepes at pH 7.4). Fractions containing A2M were pooled, concentrated by ultrafiltration, and purified by size-exclusion chromatography on a Sephacryl S-300 HR (GE Healthcare), using a 20 mM Hepes, 150 mM NaCl, pH 7.4 running buffer (Hepes-buffered saline [HBS]). Endogenous human A2M was purified from 4 to 16% PEG-cut plasma using this same protocol.

### Reaction of A2M with methylamine and proteases

To aminolyze TE domain of A2M, methylamine (pH 8) was added to 250 mM and incubated for at least 45 min at 37 °C. To assess the cleavage of A2M by bovine pancreatic trypsin (Sigma) and LysC (a.k.a. lysyl endopeptidase; FUJIFILM), proteases were added to the indicated molar ratios (typically 2.2:1 mol/mol of protease:A2M) and incubated for 5 min at 37 °C. The digestion was then inhibited using the serine protease inhibitor PMSF (2 mM, 15 min, room temperature). To assess the cleavage of A2M by MMP2, MMP2 was added to A2M, incubated for 15 min at 37 °C, and then inhibited using 20 mM EDTA. When cleaving A2M using other human proteases, incubation was for 1 h at 37 °C, and PMSF or EDTA was used to inhibit the digestion, as appropriate.

### Depletion of collapsed A2M using LRP1-conjugated resin

The A2M-binding fragment of LRP1, cluster 1B (38), was expressed with two N-terminal StrepII tags and a C-terminal Fc region from human immunoglobulin G1, and purified as previously described (35). About 600 μg LRP1 were conjugated onto 200 mg of *N*-hydroxysuccinimide-activated agarose (Pierce) in 0.15 M triethylamine bicarbonate, 0.15 M Hepes, pH 8.3, for 2 h at room temperature with mixing on a rotator. Conjugation was quenched with 50 mM Tris–HCl.

Collapsed recombinant A2M was depleted by adding 10 mM CaCl_2_ to the A2M solution and incubating it for at least 2 h with the LRP1 resin on a rotator at room temperature. The flowthrough from the resin was then saved as the depleted A2M sample, and the resin was regenerated first by eluting with three rounds of 50 mM EDTA in HBS, followed by three rounds of washing with 10 mM CaCl_2_ in HBS. The abundance of native A2M in the sample before and after depletion was then assessed by pore-limited native PAGE.

### SDS-PAGE and pore-limited native PAGE

Pore-limited native PAGE was performed as previously described (39), with homemade 5 to 10% acrylamide gradient gels and Tris/borate/EDTA running buffer. Denaturing SDS-PAGE was performed using the discontinuous 2-amino-2-methyl-1,3-propanediol and glycine buffer system with homemade 5 to 15% acrylamide gradient gels (40). SDS-PAGE samples were reduced by 25 mM DTT at 95 °C for 5 min.

### In-gel digest using pepsin and LC–MS/MS analysis

The digestion of electrophoretically separated protein samples into peptides using pepsin and analysis of the peptides by LC–MS/MS was used to quantify the TE abundance in wildtype A2M, as well as the EXT5, EXT10, EXT20, nEXT5, nEXT10, and nEXT20 mutants, after they were separated by pore-limited native PAGE. Gel bands were excised, shrunk with acetonitrile, and then swelled with 0.1% v/v acetic acid, pH 3. Shrinking and swelling were repeated twice to wash the gel bands. The gel bands were shrunk a final time, dried, and then swelled in 0.1% v/v acetic acid, pH 3 with pepsin added to a final 1:20 w/w ratio of pepsin:sample. Digestion with pepsin was carried out overnight at 37 °C. Peptides from the digested samples were then purified using pipette tips packed with POROS 50 R2 C18 resin (PerSeptive Biosystems, Inc).

Approximately 250 ng of peptide were analyzed by LC–MS/MS with an EASY-nLC 1200 (Thermo Fisher Scientific) and an Orbitrap Eclipse Tribrid mass spectrometer (Thermo Fisher Scientific). A targeted method was used for quantification of TE of A2M by PRM, continually selecting the LQMPYGCGEQN peptide (modified with the loss of ammonia, −17.026 Da) for MS2 using high-energy collision-induced dissociation.

PRM data were quantified using Skyline, version 20.1.0.155 (https://skyline.ms/project/home/begin.view?) (41), using the b10 and y2-y9 product ions from the LQMPYGCGEQN peptide, with precursor and fragment mass tolerances of 10 ppm. The raw data files and Skyline file have been made available; see Data availability section.

## Data availability

The raw mass spectrometry data files used to determine the TE abundance of A2M EXT and nEXT proteins by PRM have been deposited to the ProteomeXchange Consortium *via* the PRIDE (42) partner repository with the dataset identifier PXD023651, along with the corresponding Skyline quantification file. The full sequences of all recombinant A2M proteins used in this study are included in the supporting information.

## Supporting information

This article contains supporting information.

## Conflict of interest

The authors declare that they have no conflicts of interest with the contents of this article.
