# Supplementary material for: The conformational change of the protease inhibitor α2-macroglobulin is triggered by the retraction of the cleaved bait region from a central channel
Source: J Biol Chem. 2022 Jul 1;298(8):102230. doi: 10.1016/j.jbc.2022.102230 (PMC9352918; doi:10.1016/j.jbc.2022.102230)
Supplement: Supplemental Figures S1–S4 and Table S1 [file mmc1.docx]

**Supporting information**

**The conformational change of the protease inhibitor α_2_-macroglobulin is triggered by the retraction of the cleaved bait region from a central channel.**

Seandean Lykke Harwood, Khang Diep, Nadia Sukusu Nielsen, Kathrine Tejlgård Jensen, Jan J. Enghild

Department of Molecular Biology and Genetics, Aarhus University, Aarhus 8000, Denmark

**Supporting information in this document**

[TABLE S1. Bait region sequences of recombinant A2M proteins. 2](#_Toc94796362)

[FIGURE S1. Western blotting of A2M with EXT bait regions 3](#_Toc94796363)

[FIGURE S2. Depletion of non-native A2M using LRP1-conjugated resin 4](#_Toc94796364)

[FIGURE S3. Identifying selectively reducing concentrations of glutathione and thioredoxin 5](#_Toc94796366)

[FIGURE S4. Interactions between the α’NT region and MG7 domain 6](#_Toc94796367)

[Full sequences for all recombinant A2M proteins 7](#_Toc94796368)

**Other supporting information:**

**MS data:** The raw mass spectrometry data files used to determine the thiol ester abundance of A2M EXT and nEXT proteins by PRM have been deposited to the ProteomeXchange Consortium via the PRIDE (42) partner repository with the dataset identifier PXD023651, along with the corresponding Skyline quantification file.

## TABLE S1. Bait region sequences of recombinant A2M proteins.

The sequences of all recombinant A2M proteins with altered bait region sequences that were used in this study are given between the Cys689 and Phe735* residues. Important mutated residues are bolded. Note that some A2M mutants have bait region lengths different than wildtype, which changes Phe735’s numbering from that of wildtype.

| Name | Sequence from Cys689 to F735* |
| --- | --- |
| nEXT20 | CPQLQQYEMHGPEGLRVGFYES**GGSGGSGGSGGSGGSGGSGG**DVMGRGHARLVHVEEPHTETVRKYF |
| nEXT10 | CPQLQQYEMHGPEGLRVGFYES**GGSGGSGGSG**DVMGRGHARLVHVEEPHTETVRKYF |
| nEXT5 | CPQLQQYEMHGPEGLRVGFYES**GGSGG**DVMGRGHARLVHVEEPHTETVRKYF |
| Y734A F735A | CPQLQQYEMHGPEGLRVGFYESDVMGRGHARLVHVEEPHTETVRK**AA** |
| F735A | CPQLQQYEMHGPEGLRVGFYESDVMGRGHARLVHVEEPHTETVRKY**A** |
| ∆α'NT | CPQLQQYEMHGPEGLRVGFYESDVMGRGHARLVHVEEPHT**GGSGGS**F |
| R732A | CPQLQQYEMHGPEGLRVGFYESDVMGRGHARLVHVEEPHTETV**A**KYF |
| Wildtype | CPQLQQYEMHGPEGLRVGFYESDVMGRGHARLVHVEEPHTETVRKYF |
| EXT5 | CGGSGGSGGSGGSGG**K**GGSGGSGGSGGSGGSGGSGGSGGS**GGSGG**ETVRKYF |
| EXT10 | CGGSGGSGGSGGSGG**K**GGSGGSGGSGGSGGSGGSGGSGGS**GGSGGSGGSG**ETVRKYF |
| EXT20 | CGGSGGSGGSGGSGG**K**GGSGGSGGSGGSGGSGGSGGSGGS**GGSGGSGGSGGSGGSGGSGG**ETVRKYF |
| LOOP3 | CGGSGGSGGSG**R**S**CGKGC**S**R**GSGGSGGSGGSGGSGGSGGSETVRKYF |
| LOOP8 | CGGSGGSGG**R**G**CSGGKGGSGC**S**R**GSGGSGGSGGSGGSGGSGGSGGETVRKYF |
| 3xLOOP8 | CGGSG**CSGGKGGSGC**GGSGG**R**GGSGG**CSGGKGGSGC**GGSGG**R**GGSGG**CSGGKGGSGC**GETVRKYF |
| LOOP23 | CGGSGGSGG**R**G**CSGGKGGSGGSGGSGGSGGSGGSGC**S**R**GSGGSGGSGGSGGSGGSGGSGGETVRKYF |

**
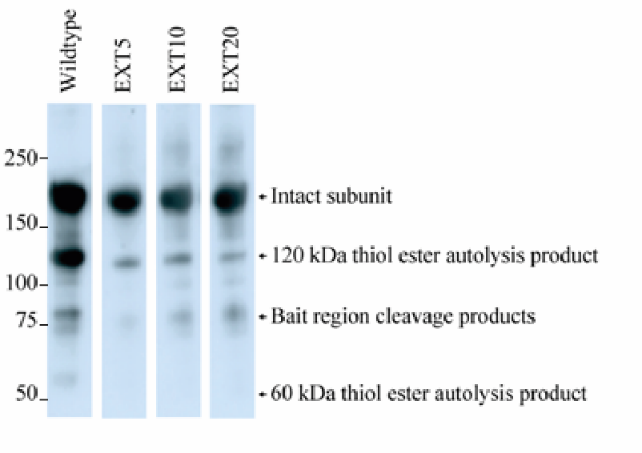
**

## FIGURE S1. Western blotting of A2M with EXT bait regions. Purified recombinant A2M with the wildtype, EXT5, EXT10, or EXT20 bait region were analyzed by reduced SDS-PAGE and Western blotting using the polyclonal rabbit anti-human A2M antibody Q010205-2 (Agilent) and a goat anti-rabbit antibody HRP conjugate. All recombinantly expressed A2Ms were mostly intact, indicating that the collapsed conformations seen for the EXT proteins was not due to bait region cleavage by cell culture proteases during their expression.


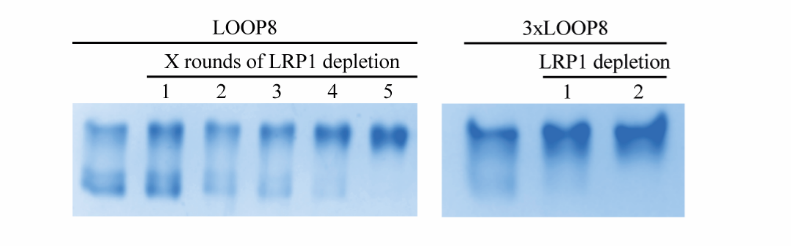


## FIGURE S2. Depletion of non-native A2M from the LOOP8 and 3xLOOP8 A2M proteins using LRP1-conjugated resin. Recombinant A2Ms with significant non-native A2M content were depleted using LRP1-conjugated resin in order to purify native, functional A2M. The A2M samples before depletion and after the indicated number of rounds of LRP1 depletion were analyzed by pore limited native PAGE to verify that non-native A2M had been removed.


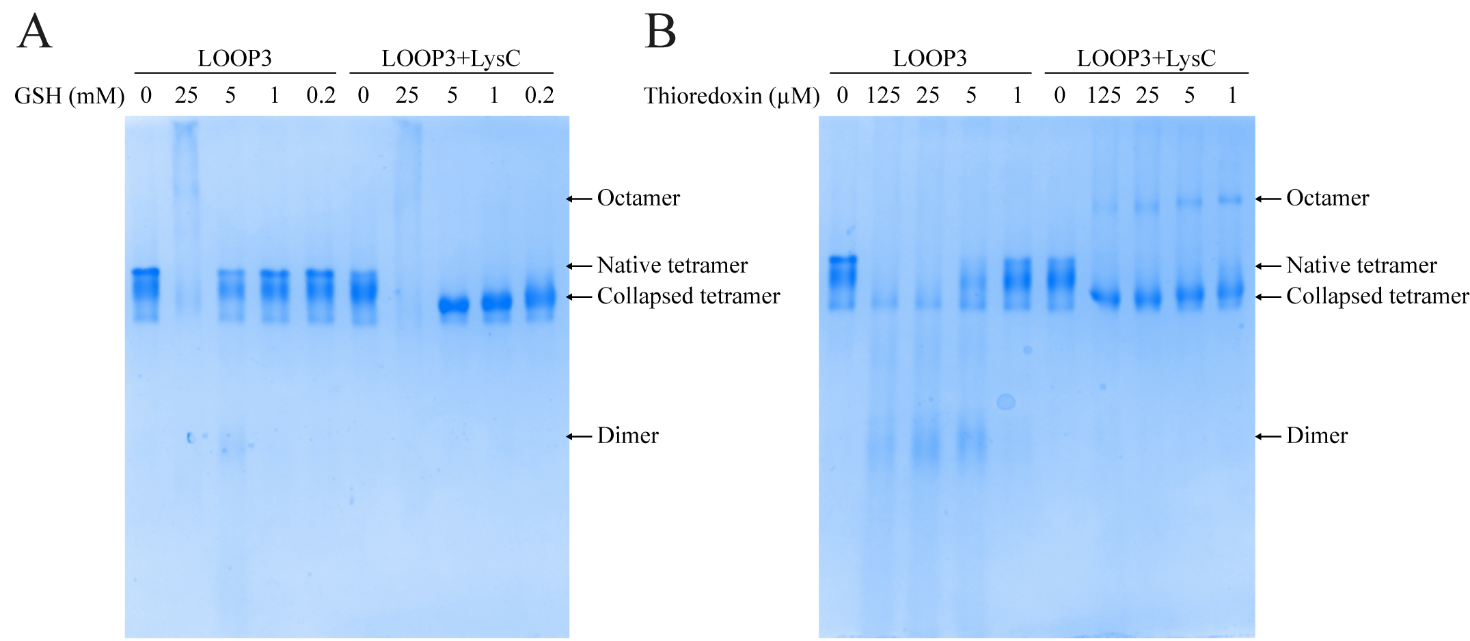


## FIGURE S3. **Identifying selectively reducing concentrations of glutathione and thioredoxin.** A2M LOOP3, with and without LysC cleavage of its disulfide-excluded lysine residue, was reduced by a titration series of glutathione (A) and thioredoxin (B). Dissociation of A2M into dimers due to the reduction of its endogenous inter-subunit disulfides was seen at high concentration of both redox reagents, although both reagents were also able to reduce the bait region disulfide and cause LysC-cleaved A2M LOOP3 to collapse. Unexpectedly, thioredoxin could also form A2M octamers, probably by bridging the cleaved bait regions of two separate A2M tetramers through disulfide formation instead of reduction. Therefore, 1 mM glutathione was chosen for subsequent experiments, as this was sufficient to reduce the bait region disulfide and collapse LysC-cleaved A2M LOOP3 without dissociating A2M into dimers.


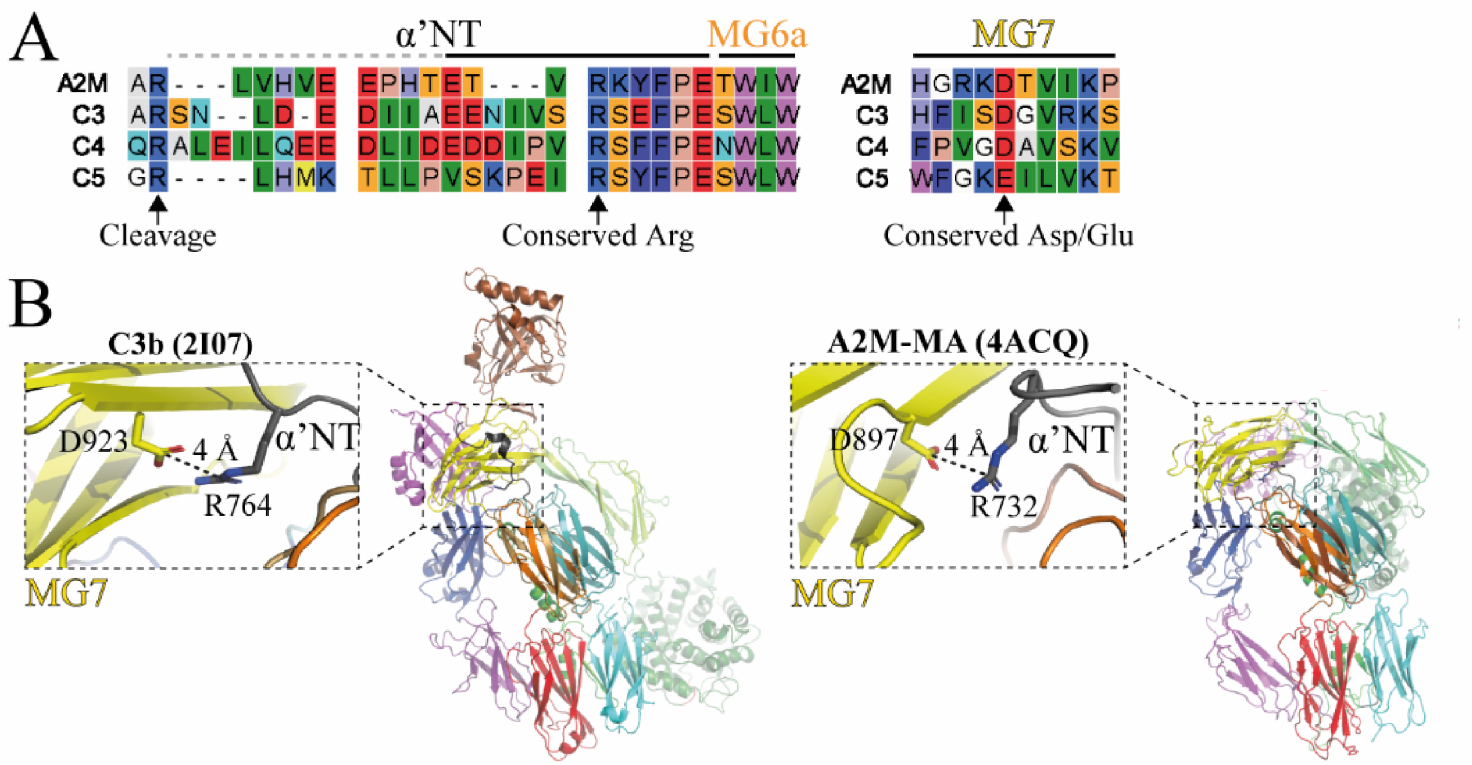
FIGURE S4. Interactions between the α’NT region and MG7 domain after the A2MF conformational change. (**A**) Alignment of the sequence of the A2M and complement factor α’NT regions and MG7 domain in the vicinity of the conserved arginine (Arg732 in A2M) and aspartate/glutamate (Asp897 in A2M). The convertase cleavage site of the complement factors is also indicated. (**B**) Structures of C3 (induced by trypsin cleavage to produce C3b) and A2M (induced by thiol ester aminolysis by methylamine) after their conformational changes, showing the interactions between the α’NT region and MG7 domain and the ionic pairing of the conserved arginine and aspartate residues.

## Full sequences for all recombinant A2M proteins

>A2M_wildtype

MGKNKLLHPSLVLLLLVLLPTDASVSGKPQYMVLVPSLLHTETTEKGCVLLSYLNETVTVSASLESVRGNRSLFTDLEAENDVLHCVAFAVPKSSSNEEVMFLTVQVKGPTQEFKKRTTVMVKNEDSLVFVQTDKSIYKPGQTVKFRVVSMDENFHPLNELIPLVYIQDPKGNRIAQWQSFQLEGGLKQFSFPLSSEPFQGSYKVVVQKKSGGRTEHPFTVEEFVLPKFEVQVTVPKIITILEEEMNVSVCGLYTYGKPVPGHVTVSICRKYSDASDCHGEDSQAFCEKFSGQLNSHGCFYQQVKTKVFQLKRKEYEMKLHTEAQIQEEGTVVELTGRQSSEITRTITKLSFVKVDSHFRQGIPFFGQVRLVDGKGVPIPNKVIFIRGNEANYYSNATTDEHGLVQFSINTTNVMGTSLTVRVNYKDRSPCYGYQWVSEEHEEAHHTAYLVFSPSKSFVHLEPMSHELPCGHTQTVQAHYILNGGTLLGLKKLSFYYLIMAKGGIVRTGTHGLLVKQEDMKGHFSISIPVKSDIAPVARLLIYAVLPTGDVIGDSAKYDVENCLANKVDLSFSPSQSLPASHAHLRVTAAPQSVCALRAVDQSVLLMKPDAELSASSVYNLLPEKDLTGFPGPLNDQDDEDCINRHNVYINGITYTPVSSTNEKDMYSFLEDMGLKAFTNSKIRKPKMCPQLQQYEMHGPEGLRVGFYESDVMGRGHARLVHVEEPHTETVRKYFPETWIWDLVVVNSAGVAEVGVTVPDTITEWKAGAFCLSEDAGLGISSTASLRAFQPFFVELTMPYSVIRGEAFTLKATVLNYLPKCIRVSVQLEASPAFLAVPVEKEQAPHCICANGRQTVSWAVTPKSLGNVNFTVSAEALESQELCGTEVPSVPEHGRKDTVIKPLLVEPEGLEKETTFNSLLCPSGGEVSEELSLKLPPNVVEESARASVSVLGDILGSAMQNTQNLLQMPYGCGEQNMVLFAPNIYVLDYLNETQQLTPEVKSKAIGYLNTGYQRQLNYKHYDGSYSTFGERYGRNQGNTWLTAFVLKTFAQARAYIFIDEAHITQALIWLSQRQKDNGCFRSSGSLLNNAIKGGVEDEVTLSAYITIALLEIPLTVTHPVVRNALFCLESAWKTAQEGDHGSHVYTKALLAYAFALAGNQDKRKEVLKSLNEEAVKKDNSVHWERPQKPKAPVGHFYEPQAPSAEVEMTSYVLLAYLTAQPAPTSEDLTSATNIVKWITKQQNAQGGFSSTQDTVVALHALSKYGAATFTRTGKAAQVTIQSSGTFSSKFQVDNNNRLLLQQVSLPELPGEYSMKVTGEGCVYLQTSLKYNILPEKEEFPFALGVQTLPQTCDEPKAHTSFQISLSVSYTGSRSASNMAIVDVKMVSGFIPLKPTVKMLERSNHVSRTEVSSNHVLIYLDKVSNQTLSLFFTVLQDVPVRDLKPAIVKVYDYYETDEFAIAEYNAPCSKDLGNA*

>A2M_nEXT5

MGKNKLLHPSLVLLLLVLLPTDASVSGKPQYMVLVPSLLHTETTEKGCVLLSYLNETVTVSASLESVRGNRSLFTDLEAENDVLHCVAFAVPKSSSNEEVMFLTVQVKGPTQEFKKRTTVMVKNEDSLVFVQTDKSIYKPGQTVKFRVVSMDENFHPLNELIPLVYIQDPKGNRIAQWQSFQLEGGLKQFSFPLSSEPFQGSYKVVVQKKSGGRTEHPFTVEEFVLPKFEVQVTVPKIITILEEEMNVSVCGLYTYGKPVPGHVTVSICRKYSDASDCHGEDSQAFCEKFSGQLNSHGCFYQQVKTKVFQLKRKEYEMKLHTEAQIQEEGTVVELTGRQSSEITRTITKLSFVKVDSHFRQGIPFFGQVRLVDGKGVPIPNKVIFIRGNEANYYSNATTDEHGLVQFSINTTNVMGTSLTVRVNYKDRSPCYGYQWVSEEHEEAHHTAYLVFSPSKSFVHLEPMSHELPCGHTQTVQAHYILNGGTLLGLKKLSFYYLIMAKGGIVRTGTHGLLVKQEDMKGHFSISIPVKSDIAPVARLLIYAVLPTGDVIGDSAKYDVENCLANKVDLSFSPSQSLPASHAHLRVTAAPQSVCALRAVDQSVLLMKPDAELSASSVYNLLPEKDLTGFPGPLNDQDDEDCINRHNVYINGITYTPVSSTNEKDMYSFLEDMGLKAFTNSKIRKPKMCPQLQQYEMHGPEGLRVGFYESGGSGGDVMGRGHARLVHVEEPHTETVRKYFPETWIWDLVVVNSAGVAEVGVTVPDTITEWKAGAFCLSEDAGLGISSTASLRAFQPFFVELTMPYSVIRGEAFTLKATVLNYLPKCIRVSVQLEASPAFLAVPVEKEQAPHCICANGRQTVSWAVTPKSLGNVNFTVSAEALESQELCGTEVPSVPEHGRKDTVIKPLLVEPEGLEKETTFNSLLCPSGGEVSEELSLKLPPNVVEESARASVSVLGDILGSAMQNTQNLLQMPYGCGEQNMVLFAPNIYVLDYLNETQQLTPEVKSKAIGYLNTGYQRQLNYKHYDGSYSTFGERYGRNQGNTWLTAFVLKTFAQARAYIFIDEAHITQALIWLSQRQKDNGCFRSSGSLLNNAIKGGVEDEVTLSAYITIALLEIPLTVTHPVVRNALFCLESAWKTAQEGDHGSHVYTKALLAYAFALAGNQDKRKEVLKSLNEEAVKKDNSVHWERPQKPKAPVGHFYEPQAPSAEVEMTSYVLLAYLTAQPAPTSEDLTSATNIVKWITKQQNAQGGFSSTQDTVVALHALSKYGAATFTRTGKAAQVTIQSSGTFSSKFQVDNNNRLLLQQVSLPELPGEYSMKVTGEGCVYLQTSLKYNILPEKEEFPFALGVQTLPQTCDEPKAHTSFQISLSVSYTGSRSASNMAIVDVKMVSGFIPLKPTVKMLERSNHVSRTEVSSNHVLIYLDKVSNQTLSLFFTVLQDVPVRDLKPAIVKVYDYYETDEFAIAEYNAPCSKDLGNA*

>A2M_nEXT10

MGKNKLLHPSLVLLLLVLLPTDASVSGKPQYMVLVPSLLHTETTEKGCVLLSYLNETVTVSASLESVRGNRSLFTDLEAENDVLHCVAFAVPKSSSNEEVMFLTVQVKGPTQEFKKRTTVMVKNEDSLVFVQTDKSIYKPGQTVKFRVVSMDENFHPLNELIPLVYIQDPKGNRIAQWQSFQLEGGLKQFSFPLSSEPFQGSYKVVVQKKSGGRTEHPFTVEEFVLPKFEVQVTVPKIITILEEEMNVSVCGLYTYGKPVPGHVTVSICRKYSDASDCHGEDSQAFCEKFSGQLNSHGCFYQQVKTKVFQLKRKEYEMKLHTEAQIQEEGTVVELTGRQSSEITRTITKLSFVKVDSHFRQGIPFFGQVRLVDGKGVPIPNKVIFIRGNEANYYSNATTDEHGLVQFSINTTNVMGTSLTVRVNYKDRSPCYGYQWVSEEHEEAHHTAYLVFSPSKSFVHLEPMSHELPCGHTQTVQAHYILNGGTLLGLKKLSFYYLIMAKGGIVRTGTHGLLVKQEDMKGHFSISIPVKSDIAPVARLLIYAVLPTGDVIGDSAKYDVENCLANKVDLSFSPSQSLPASHAHLRVTAAPQSVCALRAVDQSVLLMKPDAELSASSVYNLLPEKDLTGFPGPLNDQDDEDCINRHNVYINGITYTPVSSTNEKDMYSFLEDMGLKAFTNSKIRKPKMCPQLQQYEMHGPEGLRVGFYESGGSGGSGGSGDVMGRGHARLVHVEEPHTETVRKYFPETWIWDLVVVNSAGVAEVGVTVPDTITEWKAGAFCLSEDAGLGISSTASLRAFQPFFVELTMPYSVIRGEAFTLKATVLNYLPKCIRVSVQLEASPAFLAVPVEKEQAPHCICANGRQTVSWAVTPKSLGNVNFTVSAEALESQELCGTEVPSVPEHGRKDTVIKPLLVEPEGLEKETTFNSLLCPSGGEVSEELSLKLPPNVVEESARASVSVLGDILGSAMQNTQNLLQMPYGCGEQNMVLFAPNIYVLDYLNETQQLTPEVKSKAIGYLNTGYQRQLNYKHYDGSYSTFGERYGRNQGNTWLTAFVLKTFAQARAYIFIDEAHITQALIWLSQRQKDNGCFRSSGSLLNNAIKGGVEDEVTLSAYITIALLEIPLTVTHPVVRNALFCLESAWKTAQEGDHGSHVYTKALLAYAFALAGNQDKRKEVLKSLNEEAVKKDNSVHWERPQKPKAPVGHFYEPQAPSAEVEMTSYVLLAYLTAQPAPTSEDLTSATNIVKWITKQQNAQGGFSSTQDTVVALHALSKYGAATFTRTGKAAQVTIQSSGTFSSKFQVDNNNRLLLQQVSLPELPGEYSMKVTGEGCVYLQTSLKYNILPEKEEFPFALGVQTLPQTCDEPKAHTSFQISLSVSYTGSRSASNMAIVDVKMVSGFIPLKPTVKMLERSNHVSRTEVSSNHVLIYLDKVSNQTLSLFFTVLQDVPVRDLKPAIVKVYDYYETDEFAIAEYNAPCSKDLGNA*

>A2M_nEXT20

MGKNKLLHPSLVLLLLVLLPTDASVSGKPQYMVLVPSLLHTETTEKGCVLLSYLNETVTVSASLESVRGNRSLFTDLEAENDVLHCVAFAVPKSSSNEEVMFLTVQVKGPTQEFKKRTTVMVKNEDSLVFVQTDKSIYKPGQTVKFRVVSMDENFHPLNELIPLVYIQDPKGNRIAQWQSFQLEGGLKQFSFPLSSEPFQGSYKVVVQKKSGGRTEHPFTVEEFVLPKFEVQVTVPKIITILEEEMNVSVCGLYTYGKPVPGHVTVSICRKYSDASDCHGEDSQAFCEKFSGQLNSHGCFYQQVKTKVFQLKRKEYEMKLHTEAQIQEEGTVVELTGRQSSEITRTITKLSFVKVDSHFRQGIPFFGQVRLVDGKGVPIPNKVIFIRGNEANYYSNATTDEHGLVQFSINTTNVMGTSLTVRVNYKDRSPCYGYQWVSEEHEEAHHTAYLVFSPSKSFVHLEPMSHELPCGHTQTVQAHYILNGGTLLGLKKLSFYYLIMAKGGIVRTGTHGLLVKQEDMKGHFSISIPVKSDIAPVARLLIYAVLPTGDVIGDSAKYDVENCLANKVDLSFSPSQSLPASHAHLRVTAAPQSVCALRAVDQSVLLMKPDAELSASSVYNLLPEKDLTGFPGPLNDQDDEDCINRHNVYINGITYTPVSSTNEKDMYSFLEDMGLKAFTNSKIRKPKMCPQLQQYEMHGPEGLRVGFYESGGSGGSGGSGGSGGSGGSGGDVMGRGHARLVHVEEPHTETVRKYFPETWIWDLVVVNSAGVAEVGVTVPDTITEWKAGAFCLSEDAGLGISSTASLRAFQPFFVELTMPYSVIRGEAFTLKATVLNYLPKCIRVSVQLEASPAFLAVPVEKEQAPHCICANGRQTVSWAVTPKSLGNVNFTVSAEALESQELCGTEVPSVPEHGRKDTVIKPLLVEPEGLEKETTFNSLLCPSGGEVSEELSLKLPPNVVEESARASVSVLGDILGSAMQNTQNLLQMPYGCGEQNMVLFAPNIYVLDYLNETQQLTPEVKSKAIGYLNTGYQRQLNYKHYDGSYSTFGERYGRNQGNTWLTAFVLKTFAQARAYIFIDEAHITQALIWLSQRQKDNGCFRSSGSLLNNAIKGGVEDEVTLSAYITIALLEIPLTVTHPVVRNALFCLESAWKTAQEGDHGSHVYTKALLAYAFALAGNQDKRKEVLKSLNEEAVKKDNSVHWERPQKPKAPVGHFYEPQAPSAEVEMTSYVLLAYLTAQPAPTSEDLTSATNIVKWITKQQNAQGGFSSTQDTVVALHALSKYGAATFTRTGKAAQVTIQSSGTFSSKFQVDNNNRLLLQQVSLPELPGEYSMKVTGEGCVYLQTSLKYNILPEKEEFPFALGVQTLPQTCDEPKAHTSFQISLSVSYTGSRSASNMAIVDVKMVSGFIPLKPTVKMLERSNHVSRTEVSSNHVLIYLDKVSNQTLSLFFTVLQDVPVRDLKPAIVKVYDYYETDEFAIAEYNAPCSKDLGNA*

>A2M_EXT5

MGKNKLLHPSLVLLLLVLLPTDASVSGKPQYMVLVPSLLHTETTEKGCVLLSYLNETVTVSASLESVRGNRSLFTDLEAENDVLHCVAFAVPKSSSNEEVMFLTVQVKGPTQEFKKRTTVMVKNEDSLVFVQTDKSIYKPGQTVKFRVVSMDENFHPLNELIPLVYIQDPKGNRIAQWQSFQLEGGLKQFSFPLSSEPFQGSYKVVVQKKSGGRTEHPFTVEEFVLPKFEVQVTVPKIITILEEEMNVSVCGLYTYGKPVPGHVTVSICRKYSDASDCHGEDSQAFCEKFSGQLNSHGCFYQQVKTKVFQLKRKEYEMKLHTEAQIQEEGTVVELTGRQSSEITRTITKLSFVKVDSHFRQGIPFFGQVRLVDGKGVPIPNKVIFIRGNEANYYSNATTDEHGLVQFSINTTNVMGTSLTVRVNYKDRSPCYGYQWVSEEHEEAHHTAYLVFSPSKSFVHLEPMSHELPCGHTQTVQAHYILNGGTLLGLKKLSFYYLIMAKGGIVRTGTHGLLVKQEDMKGHFSISIPVKSDIAPVARLLIYAVLPTGDVIGDSAKYDVENCLANKVDLSFSPSQSLPASHAHLRVTAAPQSVCALRAVDQSVLLMKPDAELSASSVYNLLPEKDLTGFPGPLNDQDDEDCINRHNVYINGITYTPVSSTNEKDMYSFLEDMGLKAFTNSKIRKPKMCGGSGGSGGSGGSGGKGGSGGSGGSGGSGGSGGSGGSGGSGGSGGETVRKYFPETWIWDLVVVNSAGVAEVGVTVPDTITEWKAGAFCLSEDAGLGISSTASLRAFQPFFVELTMPYSVIRGEAFTLKATVLNYLPKCIRVSVQLEASPAFLAVPVEKEQAPHCICANGRQTVSWAVTPKSLGNVNFTVSAEALESQELCGTEVPSVPEHGRKDTVIKPLLVEPEGLEKETTFNSLLCPSGGEVSEELSLKLPPNVVEESARASVSVLGDILGSAMQNTQNLLQMPYGCGEQNMVLFAPNIYVLDYLNETQQLTPEVKSKAIGYLNTGYQRQLNYKHYDGSYSTFGERYGRNQGNTWLTAFVLKTFAQARAYIFIDEAHITQALIWLSQRQKDNGCFRSSGSLLNNAIKGGVEDEVTLSAYITIALLEIPLTVTHPVVRNALFCLESAWKTAQEGDHGSHVYTKALLAYAFALAGNQDKRKEVLKSLNEEAVKKDNSVHWERPQKPKAPVGHFYEPQAPSAEVEMTSYVLLAYLTAQPAPTSEDLTSATNIVKWITKQQNAQGGFSSTQDTVVALHALSKYGAATFTRTGKAAQVTIQSSGTFSSKFQVDNNNRLLLQQVSLPELPGEYSMKVTGEGCVYLQTSLKYNILPEKEEFPFALGVQTLPQTCDEPKAHTSFQISLSVSYTGSRSASNMAIVDVKMVSGFIPLKPTVKMLERSNHVSRTEVSSNHVLIYLDKVSNQTLSLFFTVLQDVPVRDLKPAIVKVYDYYETDEFAIAEYNAPCSKDLGNA*

>A2M_EXT10

MGKNKLLHPSLVLLLLVLLPTDASVSGKPQYMVLVPSLLHTETTEKGCVLLSYLNETVTVSASLESVRGNRSLFTDLEAENDVLHCVAFAVPKSSSNEEVMFLTVQVKGPTQEFKKRTTVMVKNEDSLVFVQTDKSIYKPGQTVKFRVVSMDENFHPLNELIPLVYIQDPKGNRIAQWQSFQLEGGLKQFSFPLSSEPFQGSYKVVVQKKSGGRTEHPFTVEEFVLPKFEVQVTVPKIITILEEEMNVSVCGLYTYGKPVPGHVTVSICRKYSDASDCHGEDSQAFCEKFSGQLNSHGCFYQQVKTKVFQLKRKEYEMKLHTEAQIQEEGTVVELTGRQSSEITRTITKLSFVKVDSHFRQGIPFFGQVRLVDGKGVPIPNKVIFIRGNEANYYSNATTDEHGLVQFSINTTNVMGTSLTVRVNYKDRSPCYGYQWVSEEHEEAHHTAYLVFSPSKSFVHLEPMSHELPCGHTQTVQAHYILNGGTLLGLKKLSFYYLIMAKGGIVRTGTHGLLVKQEDMKGHFSISIPVKSDIAPVARLLIYAVLPTGDVIGDSAKYDVENCLANKVDLSFSPSQSLPASHAHLRVTAAPQSVCALRAVDQSVLLMKPDAELSASSVYNLLPEKDLTGFPGPLNDQDDEDCINRHNVYINGITYTPVSSTNEKDMYSFLEDMGLKAFTNSKIRKPKMCGGSGGSGGSGGSGGKGGSGGSGGSGGSGGSGGSGGSGGSGGSGGSGGSGETVRKYFPETWIWDLVVVNSAGVAEVGVTVPDTITEWKAGAFCLSEDAGLGISSTASLRAFQPFFVELTMPYSVIRGEAFTLKATVLNYLPKCIRVSVQLEASPAFLAVPVEKEQAPHCICANGRQTVSWAVTPKSLGNVNFTVSAEALESQELCGTEVPSVPEHGRKDTVIKPLLVEPEGLEKETTFNSLLCPSGGEVSEELSLKLPPNVVEESARASVSVLGDILGSAMQNTQNLLQMPYGCGEQNMVLFAPNIYVLDYLNETQQLTPEVKSKAIGYLNTGYQRQLNYKHYDGSYSTFGERYGRNQGNTWLTAFVLKTFAQARAYIFIDEAHITQALIWLSQRQKDNGCFRSSGSLLNNAIKGGVEDEVTLSAYITIALLEIPLTVTHPVVRNALFCLESAWKTAQEGDHGSHVYTKALLAYAFALAGNQDKRKEVLKSLNEEAVKKDNSVHWERPQKPKAPVGHFYEPQAPSAEVEMTSYVLLAYLTAQPAPTSEDLTSATNIVKWITKQQNAQGGFSSTQDTVVALHALSKYGAATFTRTGKAAQVTIQSSGTFSSKFQVDNNNRLLLQQVSLPELPGEYSMKVTGEGCVYLQTSLKYNILPEKEEFPFALGVQTLPQTCDEPKAHTSFQISLSVSYTGSRSASNMAIVDVKMVSGFIPLKPTVKMLERSNHVSRTEVSSNHVLIYLDKVSNQTLSLFFTVLQDVPVRDLKPAIVKVYDYYETDEFAIAEYNAPCSKDLGNA*

>A2M_EXT20

MGKNKLLHPSLVLLLLVLLPTDASVSGKPQYMVLVPSLLHTETTEKGCVLLSYLNETVTVSASLESVRGNRSLFTDLEAENDVLHCVAFAVPKSSSNEEVMFLTVQVKGPTQEFKKRTTVMVKNEDSLVFVQTDKSIYKPGQTVKFRVVSMDENFHPLNELIPLVYIQDPKGNRIAQWQSFQLEGGLKQFSFPLSSEPFQGSYKVVVQKKSGGRTEHPFTVEEFVLPKFEVQVTVPKIITILEEEMNVSVCGLYTYGKPVPGHVTVSICRKYSDASDCHGEDSQAFCEKFSGQLNSHGCFYQQVKTKVFQLKRKEYEMKLHTEAQIQEEGTVVELTGRQSSEITRTITKLSFVKVDSHFRQGIPFFGQVRLVDGKGVPIPNKVIFIRGNEANYYSNATTDEHGLVQFSINTTNVMGTSLTVRVNYKDRSPCYGYQWVSEEHEEAHHTAYLVFSPSKSFVHLEPMSHELPCGHTQTVQAHYILNGGTLLGLKKLSFYYLIMAKGGIVRTGTHGLLVKQEDMKGHFSISIPVKSDIAPVARLLIYAVLPTGDVIGDSAKYDVENCLANKVDLSFSPSQSLPASHAHLRVTAAPQSVCALRAVDQSVLLMKPDAELSASSVYNLLPEKDLTGFPGPLNDQDDEDCINRHNVYINGITYTPVSSTNEKDMYSFLEDMGLKAFTNSKIRKPKMCGGSGGSGGSGGSGGKGGSGGSGGSGGSGGSGGSGGSGGSGGSGGSGGSGGSGGSGGSGGETVRKYFPETWIWDLVVVNSAGVAEVGVTVPDTITEWKAGAFCLSEDAGLGISSTASLRAFQPFFVELTMPYSVIRGEAFTLKATVLNYLPKCIRVSVQLEASPAFLAVPVEKEQAPHCICANGRQTVSWAVTPKSLGNVNFTVSAEALESQELCGTEVPSVPEHGRKDTVIKPLLVEPEGLEKETTFNSLLCPSGGEVSEELSLKLPPNVVEESARASVSVLGDILGSAMQNTQNLLQMPYGCGEQNMVLFAPNIYVLDYLNETQQLTPEVKSKAIGYLNTGYQRQLNYKHYDGSYSTFGERYGRNQGNTWLTAFVLKTFAQARAYIFIDEAHITQALIWLSQRQKDNGCFRSSGSLLNNAIKGGVEDEVTLSAYITIALLEIPLTVTHPVVRNALFCLESAWKTAQEGDHGSHVYTKALLAYAFALAGNQDKRKEVLKSLNEEAVKKDNSVHWERPQKPKAPVGHFYEPQAPSAEVEMTSYVLLAYLTAQPAPTSEDLTSATNIVKWITKQQNAQGGFSSTQDTVVALHALSKYGAATFTRTGKAAQVTIQSSGTFSSKFQVDNNNRLLLQQVSLPELPGEYSMKVTGEGCVYLQTSLKYNILPEKEEFPFALGVQTLPQTCDEPKAHTSFQISLSVSYTGSRSASNMAIVDVKMVSGFIPLKPTVKMLERSNHVSRTEVSSNHVLIYLDKVSNQTLSLFFTVLQDVPVRDLKPAIVKVYDYYETDEFAIAEYNAPCSKDLGNA*

>A2M_LOOP3

MGKNKLLHPSLVLLLLVLLPTDASVSGKPQYMVLVPSLLHTETTEKGCVLLSYLNETVTVSASLESVRGNRSLFTDLEAENDVLHCVAFAVPKSSSNEEVMFLTVQVKGPTQEFKKRTTVMVKNEDSLVFVQTDKSIYKPGQTVKFRVVSMDENFHPLNELIPLVYIQDPKGNRIAQWQSFQLEGGLKQFSFPLSSEPFQGSYKVVVQKKSGGRTEHPFTVEEFVLPKFEVQVTVPKIITILEEEMNVSVCGLYTYGKPVPGHVTVSICRKYSDASDCHGEDSQAFCEKFSGQLNSHGCFYQQVKTKVFQLKRKEYEMKLHTEAQIQEEGTVVELTGRQSSEITRTITKLSFVKVDSHFRQGIPFFGQVRLVDGKGVPIPNKVIFIRGNEANYYSNATTDEHGLVQFSINTTNVMGTSLTVRVNYKDRSPCYGYQWVSEEHEEAHHTAYLVFSPSKSFVHLEPMSHELPCGHTQTVQAHYILNGGTLLGLKKLSFYYLIMAKGGIVRTGTHGLLVKQEDMKGHFSISIPVKSDIAPVARLLIYAVLPTGDVIGDSAKYDVENCLANKVDLSFSPSQSLPASHAHLRVTAAPQSVCALRAVDQSVLLMKPDAELSASSVYNLLPEKDLTGFPGPLNDQDDEDCINRHNVYINGITYTPVSSTNEKDMYSFLEDMGLKAFTNSKIRKPKMCGGSGGSGGSGRSCGKGCSRGSGGSGGSGGSGGSGGSGGSETVRKYFPETWIWDLVVVNSAGVAEVGVTVPDTITEWKAGAFCLSEDAGLGISSTASLRAFQPFFVELTMPYSVIRGEAFTLKATVLNYLPKCIRVSVQLEASPAFLAVPVEKEQAPHCICANGRQTVSWAVTPKSLGNVNFTVSAEALESQELCGTEVPSVPEHGRKDTVIKPLLVEPEGLEKETTFNSLLCPSGGEVSEELSLKLPPNVVEESARASVSVLGDILGSAMQNTQNLLQMPYGCGEQNMVLFAPNIYVLDYLNETQQLTPEVKSKAIGYLNTGYQRQLNYKHYDGSYSTFGERYGRNQGNTWLTAFVLKTFAQARAYIFIDEAHITQALIWLSQRQKDNGCFRSSGSLLNNAIKGGVEDEVTLSAYITIALLEIPLTVTHPVVRNALFCLESAWKTAQEGDHGSHVYTKALLAYAFALAGNQDKRKEVLKSLNEEAVKKDNSVHWERPQKPKAPVGHFYEPQAPSAEVEMTSYVLLAYLTAQPAPTSEDLTSATNIVKWITKQQNAQGGFSSTQDTVVALHALSKYGAATFTRTGKAAQVTIQSSGTFSSKFQVDNNNRLLLQQVSLPELPGEYSMKVTGEGCVYLQTSLKYNILPEKEEFPFALGVQTLPQTCDEPKAHTSFQISLSVSYTGSRSASNMAIVDVKMVSGFIPLKPTVKMLERSNHVSRTEVSSNHVLIYLDKVSNQTLSLFFTVLQDVPVRDLKPAIVKVYDYYETDEFAIAEYNAPCSKDLGNA*

>A2M_LOOP8

MGKNKLLHPSLVLLLLVLLPTDASVSGKPQYMVLVPSLLHTETTEKGCVLLSYLNETVTVSASLESVRGNRSLFTDLEAENDVLHCVAFAVPKSSSNEEVMFLTVQVKGPTQEFKKRTTVMVKNEDSLVFVQTDKSIYKPGQTVKFRVVSMDENFHPLNELIPLVYIQDPKGNRIAQWQSFQLEGGLKQFSFPLSSEPFQGSYKVVVQKKSGGRTEHPFTVEEFVLPKFEVQVTVPKIITILEEEMNVSVCGLYTYGKPVPGHVTVSICRKYSDASDCHGEDSQAFCEKFSGQLNSHGCFYQQVKTKVFQLKRKEYEMKLHTEAQIQEEGTVVELTGRQSSEITRTITKLSFVKVDSHFRQGIPFFGQVRLVDGKGVPIPNKVIFIRGNEANYYSNATTDEHGLVQFSINTTNVMGTSLTVRVNYKDRSPCYGYQWVSEEHEEAHHTAYLVFSPSKSFVHLEPMSHELPCGHTQTVQAHYILNGGTLLGLKKLSFYYLIMAKGGIVRTGTHGLLVKQEDMKGHFSISIPVKSDIAPVARLLIYAVLPTGDVIGDSAKYDVENCLANKVDLSFSPSQSLPASHAHLRVTAAPQSVCALRAVDQSVLLMKPDAELSASSVYNLLPEKDLTGFPGPLNDQDDEDCINRHNVYINGITYTPVSSTNEKDMYSFLEDMGLKAFTNSKIRKPKMCGGSGGSGGRGCSGGKGGSGCSRGSGGSGGSGGSGGSGGSGGSGGETVRKYFPETWIWDLVVVNSAGVAEVGVTVPDTITEWKAGAFCLSEDAGLGISSTASLRAFQPFFVELTMPYSVIRGEAFTLKATVLNYLPKCIRVSVQLEASPAFLAVPVEKEQAPHCICANGRQTVSWAVTPKSLGNVNFTVSAEALESQELCGTEVPSVPEHGRKDTVIKPLLVEPEGLEKETTFNSLLCPSGGEVSEELSLKLPPNVVEESARASVSVLGDILGSAMQNTQNLLQMPYGCGEQNMVLFAPNIYVLDYLNETQQLTPEVKSKAIGYLNTGYQRQLNYKHYDGSYSTFGERYGRNQGNTWLTAFVLKTFAQARAYIFIDEAHITQALIWLSQRQKDNGCFRSSGSLLNNAIKGGVEDEVTLSAYITIALLEIPLTVTHPVVRNALFCLESAWKTAQEGDHGSHVYTKALLAYAFALAGNQDKRKEVLKSLNEEAVKKDNSVHWERPQKPKAPVGHFYEPQAPSAEVEMTSYVLLAYLTAQPAPTSEDLTSATNIVKWITKQQNAQGGFSSTQDTVVALHALSKYGAATFTRTGKAAQVTIQSSGTFSSKFQVDNNNRLLLQQVSLPELPGEYSMKVTGEGCVYLQTSLKYNILPEKEEFPFALGVQTLPQTCDEPKAHTSFQISLSVSYTGSRSASNMAIVDVKMVSGFIPLKPTVKMLERSNHVSRTEVSSNHVLIYLDKVSNQTLSLFFTVLQDVPVRDLKPAIVKVYDYYETDEFAIAEYNAPCSKDLGNA*

>A2M_3xLOOP8

MGKNKLLHPSLVLLLLVLLPTDASVSGKPQYMVLVPSLLHTETTEKGCVLLSYLNETVTVSASLESVRGNRSLFTDLEAENDVLHCVAFAVPKSSSNEEVMFLTVQVKGPTQEFKKRTTVMVKNEDSLVFVQTDKSIYKPGQTVKFRVVSMDENFHPLNELIPLVYIQDPKGNRIAQWQSFQLEGGLKQFSFPLSSEPFQGSYKVVVQKKSGGRTEHPFTVEEFVLPKFEVQVTVPKIITILEEEMNVSVCGLYTYGKPVPGHVTVSICRKYSDASDCHGEDSQAFCEKFSGQLNSHGCFYQQVKTKVFQLKRKEYEMKLHTEAQIQEEGTVVELTGRQSSEITRTITKLSFVKVDSHFRQGIPFFGQVRLVDGKGVPIPNKVIFIRGNEANYYSNATTDEHGLVQFSINTTNVMGTSLTVRVNYKDRSPCYGYQWVSEEHEEAHHTAYLVFSPSKSFVHLEPMSHELPCGHTQTVQAHYILNGGTLLGLKKLSFYYLIMAKGGIVRTGTHGLLVKQEDMKGHFSISIPVKSDIAPVARLLIYAVLPTGDVIGDSAKYDVENCLANKVDLSFSPSQSLPASHAHLRVTAAPQSVCALRAVDQSVLLMKPDAELSASSVYNLLPEKDLTGFPGPLNDQDDEDCINRHNVYINGITYTPVSSTNEKDMYSFLEDMGLKAFTNSKIRKPKMCGGSGCSGGKGGSGCGGSGGRGGSGGCSGGKGGSGCGGSGGRGGSGGCSGGKGGSGCGETVRKYFPETWIWDLVVVNSAGVAEVGVTVPDTITEWKAGAFCLSEDAGLGISSTASLRAFQPFFVELTMPYSVIRGEAFTLKATVLNYLPKCIRVSVQLEASPAFLAVPVEKEQAPHCICANGRQTVSWAVTPKSLGNVNFTVSAEALESQELCGTEVPSVPEHGRKDTVIKPLLVEPEGLEKETTFNSLLCPSGGEVSEELSLKLPPNVVEESARASVSVLGDILGSAMQNTQNLLQMPYGCGEQNMVLFAPNIYVLDYLNETQQLTPEVKSKAIGYLNTGYQRQLNYKHYDGSYSTFGERYGRNQGNTWLTAFVLKTFAQARAYIFIDEAHITQALIWLSQRQKDNGCFRSSGSLLNNAIKGGVEDEVTLSAYITIALLEIPLTVTHPVVRNALFCLESAWKTAQEGDHGSHVYTKALLAYAFALAGNQDKRKEVLKSLNEEAVKKDNSVHWERPQKPKAPVGHFYEPQAPSAEVEMTSYVLLAYLTAQPAPTSEDLTSATNIVKWITKQQNAQGGFSSTQDTVVALHALSKYGAATFTRTGKAAQVTIQSSGTFSSKFQVDNNNRLLLQQVSLPELPGEYSMKVTGEGCVYLQTSLKYNILPEKEEFPFALGVQTLPQTCDEPKAHTSFQISLSVSYTGSRSASNMAIVDVKMVSGFIPLKPTVKMLERSNHVSRTEVSSNHVLIYLDKVSNQTLSLFFTVLQDVPVRDLKPAIVKVYDYYETDEFAIAEYNAPCSKDLGNA*

>A2M_LOOP23

MGKNKLLHPSLVLLLLVLLPTDASVSGKPQYMVLVPSLLHTETTEKGCVLLSYLNETVTVSASLESVRGNRSLFTDLEAENDVLHCVAFAVPKSSSNEEVMFLTVQVKGPTQEFKKRTTVMVKNEDSLVFVQTDKSIYKPGQTVKFRVVSMDENFHPLNELIPLVYIQDPKGNRIAQWQSFQLEGGLKQFSFPLSSEPFQGSYKVVVQKKSGGRTEHPFTVEEFVLPKFEVQVTVPKIITILEEEMNVSVCGLYTYGKPVPGHVTVSICRKYSDASDCHGEDSQAFCEKFSGQLNSHGCFYQQVKTKVFQLKRKEYEMKLHTEAQIQEEGTVVELTGRQSSEITRTITKLSFVKVDSHFRQGIPFFGQVRLVDGKGVPIPNKVIFIRGNEANYYSNATTDEHGLVQFSINTTNVMGTSLTVRVNYKDRSPCYGYQWVSEEHEEAHHTAYLVFSPSKSFVHLEPMSHELPCGHTQTVQAHYILNGGTLLGLKKLSFYYLIMAKGGIVRTGTHGLLVKQEDMKGHFSISIPVKSDIAPVARLLIYAVLPTGDVIGDSAKYDVENCLANKVDLSFSPSQSLPASHAHLRVTAAPQSVCALRAVDQSVLLMKPDAELSASSVYNLLPEKDLTGFPGPLNDQDDEDCINRHNVYINGITYTPVSSTNEKDMYSFLEDMGLKAFTNSKIRKPKMCGGSGGSGGRGCSGGKGGSGGSGGSGGSGGSGGSGCSRGSGGSGGSGGSGGSGGSGGSGGETVRKYFPETWIWDLVVVNSAGVAEVGVTVPDTITEWKAGAFCLSEDAGLGISSTASLRAFQPFFVELTMPYSVIRGEAFTLKATVLNYLPKCIRVSVQLEASPAFLAVPVEKEQAPHCICANGRQTVSWAVTPKSLGNVNFTVSAEALESQELCGTEVPSVPEHGRKDTVIKPLLVEPEGLEKETTFNSLLCPSGGEVSEELSLKLPPNVVEESARASVSVLGDILGSAMQNTQNLLQMPYGCGEQNMVLFAPNIYVLDYLNETQQLTPEVKSKAIGYLNTGYQRQLNYKHYDGSYSTFGERYGRNQGNTWLTAFVLKTFAQARAYIFIDEAHITQALIWLSQRQKDNGCFRSSGSLLNNAIKGGVEDEVTLSAYITIALLEIPLTVTHPVVRNALFCLESAWKTAQEGDHGSHVYTKALLAYAFALAGNQDKRKEVLKSLNEEAVKKDNSVHWERPQKPKAPVGHFYEPQAPSAEVEMTSYVLLAYLTAQPAPTSEDLTSATNIVKWITKQQNAQGGFSSTQDTVVALHALSKYGAATFTRTGKAAQVTIQSSGTFSSKFQVDNNNRLLLQQVSLPELPGEYSMKVTGEGCVYLQTSLKYNILPEKEEFPFALGVQTLPQTCDEPKAHTSFQISLSVSYTGSRSASNMAIVDVKMVSGFIPLKPTVKMLERSNHVSRTEVSSNHVLIYLDKVSNQTLSLFFTVLQDVPVRDLKPAIVKVYDYYETDEFAIAEYNAPCSKDLGNA*

>A2M_R732A

MGKNKLLHPSLVLLLLVLLPTDASVSGKPQYMVLVPSLLHTETTEKGCVLLSYLNETVTVSASLESVRGNRSLFTDLEAENDVLHCVAFAVPKSSSNEEVMFLTVQVKGPTQEFKKRTTVMVKNEDSLVFVQTDKSIYKPGQTVKFRVVSMDENFHPLNELIPLVYIQDPKGNRIAQWQSFQLEGGLKQFSFPLSSEPFQGSYKVVVQKKSGGRTEHPFTVEEFVLPKFEVQVTVPKIITILEEEMNVSVCGLYTYGKPVPGHVTVSICRKYSDASDCHGEDSQAFCEKFSGQLNSHGCFYQQVKTKVFQLKRKEYEMKLHTEAQIQEEGTVVELTGRQSSEITRTITKLSFVKVDSHFRQGIPFFGQVRLVDGKGVPIPNKVIFIRGNEANYYSNATTDEHGLVQFSINTTNVMGTSLTVRVNYKDRSPCYGYQWVSEEHEEAHHTAYLVFSPSKSFVHLEPMSHELPCGHTQTVQAHYILNGGTLLGLKKLSFYYLIMAKGGIVRTGTHGLLVKQEDMKGHFSISIPVKSDIAPVARLLIYAVLPTGDVIGDSAKYDVENCLANKVDLSFSPSQSLPASHAHLRVTAAPQSVCALRAVDQSVLLMKPDAELSASSVYNLLPEKDLTGFPGPLNDQDDEDCINRHNVYINGITYTPVSSTNEKDMYSFLEDMGLKAFTNSKIRKPKMCPQLQQYEMHGPEGLRVGFYESDVMGRGHARLVHVEEPHTETVAKYFPETWIWDLVVVNSAGVAEVGVTVPDTITEWKAGAFCLSEDAGLGISSTASLRAFQPFFVELTMPYSVIRGEAFTLKATVLNYLPKCIRVSVQLEASPAFLAVPVEKEQAPHCICANGRQTVSWAVTPKSLGNVNFTVSAEALESQELCGTEVPSVPEHGRKDTVIKPLLVEPEGLEKETTFNSLLCPSGGEVSEELSLKLPPNVVEESARASVSVLGDILGSAMQNTQNLLQMPYGCGEQNMVLFAPNIYVLDYLNETQQLTPEVKSKAIGYLNTGYQRQLNYKHYDGSYSTFGERYGRNQGNTWLTAFVLKTFAQARAYIFIDEAHITQALIWLSQRQKDNGCFRSSGSLLNNAIKGGVEDEVTLSAYITIALLEIPLTVTHPVVRNALFCLESAWKTAQEGDHGSHVYTKALLAYAFALAGNQDKRKEVLKSLNEEAVKKDNSVHWERPQKPKAPVGHFYEPQAPSAEVEMTSYVLLAYLTAQPAPTSEDLTSATNIVKWITKQQNAQGGFSSTQDTVVALHALSKYGAATFTRTGKAAQVTIQSSGTFSSKFQVDNNNRLLLQQVSLPELPGEYSMKVTGEGCVYLQTSLKYNILPEKEEFPFALGVQTLPQTCDEPKAHTSFQISLSVSYTGSRSASNMAIVDVKMVSGFIPLKPTVKMLERSNHVSRTEVSSNHVLIYLDKVSNQTLSLFFTVLQDVPVRDLKPAIVKVYDYYETDEFAIAEYNAPCSKDLGNA*

>A2M_∆729-734

MGKNKLLHPSLVLLLLVLLPTDASVSGKPQYMVLVPSLLHTETTEKGCVLLSYLNETVTVSASLESVRGNRSLFTDLEAENDVLHCVAFAVPKSSSNEEVMFLTVQVKGPTQEFKKRTTVMVKNEDSLVFVQTDKSIYKPGQTVKFRVVSMDENFHPLNELIPLVYIQDPKGNRIAQWQSFQLEGGLKQFSFPLSSEPFQGSYKVVVQKKSGGRTEHPFTVEEFVLPKFEVQVTVPKIITILEEEMNVSVCGLYTYGKPVPGHVTVSICRKYSDASDCHGEDSQAFCEKFSGQLNSHGCFYQQVKTKVFQLKRKEYEMKLHTEAQIQEEGTVVELTGRQSSEITRTITKLSFVKVDSHFRQGIPFFGQVRLVDGKGVPIPNKVIFIRGNEANYYSNATTDEHGLVQFSINTTNVMGTSLTVRVNYKDRSPCYGYQWVSEEHEEAHHTAYLVFSPSKSFVHLEPMSHELPCGHTQTVQAHYILNGGTLLGLKKLSFYYLIMAKGGIVRTGTHGLLVKQEDMKGHFSISIPVKSDIAPVARLLIYAVLPTGDVIGDSAKYDVENCLANKVDLSFSPSQSLPASHAHLRVTAAPQSVCALRAVDQSVLLMKPDAELSASSVYNLLPEKDLTGFPGPLNDQDDEDCINRHNVYINGITYTPVSSTNEKDMYSFLEDMGLKAFTNSKIRKPKMCPQLQQYEMHGPEGLRVGFYESDVMGRGHARLVHVEEPHTGGSGGSFPETWIWDLVVVNSAGVAEVGVTVPDTITEWKAGAFCLSEDAGLGISSTASLRAFQPFFVELTMPYSVIRGEAFTLKATVLNYLPKCIRVSVQLEASPAFLAVPVEKEQAPHCICANGRQTVSWAVTPKSLGNVNFTVSAEALESQELCGTEVPSVPEHGRKDTVIKPLLVEPEGLEKETTFNSLLCPSGGEVSEELSLKLPPNVVEESARASVSVLGDILGSAMQNTQNLLQMPYGCGEQNMVLFAPNIYVLDYLNETQQLTPEVKSKAIGYLNTGYQRQLNYKHYDGSYSTFGERYGRNQGNTWLTAFVLKTFAQARAYIFIDEAHITQALIWLSQRQKDNGCFRSSGSLLNNAIKGGVEDEVTLSAYITIALLEIPLTVTHPVVRNALFCLESAWKTAQEGDHGSHVYTKALLAYAFALAGNQDKRKEVLKSLNEEAVKKDNSVHWERPQKPKAPVGHFYEPQAPSAEVEMTSYVLLAYLTAQPAPTSEDLTSATNIVKWITKQQNAQGGFSSTQDTVVALHALSKYGAATFTRTGKAAQVTIQSSGTFSSKFQVDNNNRLLLQQVSLPELPGEYSMKVTGEGCVYLQTSLKYNILPEKEEFPFALGVQTLPQTCDEPKAHTSFQISLSVSYTGSRSASNMAIVDVKMVSGFIPLKPTVKMLERSNHVSRTEVSSNHVLIYLDKVSNQTLSLFFTVLQDVPVRDLKPAIVKVYDYYETDEFAIAEYNAPCSKDLGNA*

> A2M F735A

MGKNKLLHPSLVLLLLVLLPTDASVSGKPQYMVLVPSLLHTETTEKGCVLLSYLNETVTVSASLESVRGNRSLFTDLEAENDVLHCVAFAVPKSSSNEEVMFLTVQVKGPTQEFKKRTTVMVKNEDSLVFVQTDKSIYKPGQTVKFRVVSMDENFHPLNELIPLVYIQDPKGNRIAQWQSFQLEGGLKQFSFPLSSEPFQGSYKVVVQKKSGGRTEHPFTVEEFVLPKFEVQVTVPKIITILEEEMNVSVCGLYTYGKPVPGHVTVSICRKYSDASDCHGEDSQAFCEKFSGQLNSHGCFYQQVKTKVFQLKRKEYEMKLHTEAQIQEEGTVVELTGRQSSEITRTITKLSFVKVDSHFRQGIPFFGQVRLVDGKGVPIPNKVIFIRGNEANYYSNATTDEHGLVQFSINTTNVMGTSLTVRVNYKDRSPCYGYQWVSEEHEEAHHTAYLVFSPSKSFVHLEPMSHELPCGHTQTVQAHYILNGGTLLGLKKLSFYYLIMAKGGIVRTGTHGLLVKQEDMKGHFSISIPVKSDIAPVARLLIYAVLPTGDVIGDSAKYDVENCLANKVDLSFSPSQSLPASHAHLRVTAAPQSVCALRAVDQSVLLMKPDAELSASSVYNLLPEKDLTGFPGPLNDQDDEDCINRHNVYINGITYTPVSSTNEKDMYSFLEDMGLKAFTNSKIRKPKMCPQLQQYEMHGPEGLRVGFYESDVMGRGHARLVHVEEPHTETVRKYAPETWIWDLVVVNSAGVAEVGVTVPDTITEWKAGAFCLSEDAGLGISSTASLRAFQPFFVELTMPYSVIRGEAFTLKATVLNYLPKCIRVSVQLEASPAFLAVPVEKEQAPHCICANGRQTVSWAVTPKSLGNVNFTVSAEALESQELCGTEVPSVPEHGRKDTVIKPLLVEPEGLEKETTFNSLLCPSGGEVSEELSLKLPPNVVEESARASVSVLGDILGSAMQNTQNLLQMPYGCGEQNMVLFAPNIYVLDYLNETQQLTPEVKSKAIGYLNTGYQRQLNYKHYDGSYSTFGERYGRNQGNTWLTAFVLKTFAQARAYIFIDEAHITQALIWLSQRQKDNGCFRSSGSLLNNAIKGGVEDEVTLSAYITIALLEIPLTVTHPVVRNALFCLESAWKTAQEGDHGSHVYTKALLAYAFALAGNQDKRKEVLKSLNEEAVKKDNSVHWERPQKPKAPVGHFYEPQAPSAEVEMTSYVLLAYLTAQPAPTSEDLTSATNIVKWITKQQNAQGGFSSTQDTVVALHALSKYGAATFTRTGKAAQVTIQSSGTFSSKFQVDNNNRLLLQQVSLPELPGEYSMKVTGEGCVYLQTSLKYNILPEKEEFPFALGVQTLPQTCDEPKAHTSFQISLSVSYTGSRSASNMAIVDVKMVSGFIPLKPTVKMLERSNHVSRTEVSSNHVLIYLDKVSNQTLSLFFTVLQDVPVRDLKPAIVKVYDYYETDEFAIAEYNAPCSKDLGNA*

> A2M Y734A F735A

MGKNKLLHPSLVLLLLVLLPTDASVSGKPQYMVLVPSLLHTETTEKGCVLLSYLNETVTVSASLESVRGNRSLFTDLEAENDVLHCVAFAVPKSSSNEEVMFLTVQVKGPTQEFKKRTTVMVKNEDSLVFVQTDKSIYKPGQTVKFRVVSMDENFHPLNELIPLVYIQDPKGNRIAQWQSFQLEGGLKQFSFPLSSEPFQGSYKVVVQKKSGGRTEHPFTVEEFVLPKFEVQVTVPKIITILEEEMNVSVCGLYTYGKPVPGHVTVSICRKYSDASDCHGEDSQAFCEKFSGQLNSHGCFYQQVKTKVFQLKRKEYEMKLHTEAQIQEEGTVVELTGRQSSEITRTITKLSFVKVDSHFRQGIPFFGQVRLVDGKGVPIPNKVIFIRGNEANYYSNATTDEHGLVQFSINTTNVMGTSLTVRVNYKDRSPCYGYQWVSEEHEEAHHTAYLVFSPSKSFVHLEPMSHELPCGHTQTVQAHYILNGGTLLGLKKLSFYYLIMAKGGIVRTGTHGLLVKQEDMKGHFSISIPVKSDIAPVARLLIYAVLPTGDVIGDSAKYDVENCLANKVDLSFSPSQSLPASHAHLRVTAAPQSVCALRAVDQSVLLMKPDAELSASSVYNLLPEKDLTGFPGPLNDQDDEDCINRHNVYINGITYTPVSSTNEKDMYSFLEDMGLKAFTNSKIRKPKMCPQLQQYEMHGPEGLRVGFYESDVMGRGHARLVHVEEPHTETVRKAAPETWIWDLVVVNSAGVAEVGVTVPDTITEWKAGAFCLSEDAGLGISSTASLRAFQPFFVELTMPYSVIRGEAFTLKATVLNYLPKCIRVSVQLEASPAFLAVPVEKEQAPHCICANGRQTVSWAVTPKSLGNVNFTVSAEALESQELCGTEVPSVPEHGRKDTVIKPLLVEPEGLEKETTFNSLLCPSGGEVSEELSLKLPPNVVEESARASVSVLGDILGSAMQNTQNLLQMPYGCGEQNMVLFAPNIYVLDYLNETQQLTPEVKSKAIGYLNTGYQRQLNYKHYDGSYSTFGERYGRNQGNTWLTAFVLKTFAQARAYIFIDEAHITQALIWLSQRQKDNGCFRSSGSLLNNAIKGGVEDEVTLSAYITIALLEIPLTVTHPVVRNALFCLESAWKTAQEGDHGSHVYTKALLAYAFALAGNQDKRKEVLKSLNEEAVKKDNSVHWERPQKPKAPVGHFYEPQAPSAEVEMTSYVLLAYLTAQPAPTSEDLTSATNIVKWITKQQNAQGGFSSTQDTVVALHALSKYGAATFTRTGKAAQVTIQSSGTFSSKFQVDNNNRLLLQQVSLPELPGEYSMKVTGEGCVYLQTSLKYNILPEKEEFPFALGVQTLPQTCDEPKAHTSFQISLSVSYTGSRSASNMAIVDVKMVSGFIPLKPTVKMLERSNHVSRTEVSSNHVLIYLDKVSNQTLSLFFTVLQDVPVRDLKPAIVKVYDYYETDEFAIAEYNAPCSKDLGNA*

>A2M F224A V225G L226S

MGKNKLLHPSLVLLLLVLLPTDASVSGKPQYMVLVPSLLHTETTEKGCVLLSYLNETVTVSASLESVRGNRSLFTDLEAENDVLHCVAFAVPKSSSNEEVMFLTVQVKGPTQEFKKRTTVMVKNEDSLVFVQTDKSIYKPGQTVKFRVVSMDENFHPLNELIPLVYIQDPKGNRIAQWQSFQLEGGLKQFSFPLSSEPFQGSYKVVVQKKSGGRTEHPFTVEEAGSPKFEVQVTVPKIITILEEEMNVSVCGLYTYGKPVPGHVTVSICRKYSDASDCHGEDSQAFCEKFSGQLNSHGCFYQQVKTKVFQLKRKEYEMKLHTEAQIQEEGTVVELTGRQSSEITRTITKLSFVKVDSHFRQGIPFFGQVRLVDGKGVPIPNKVIFIRGNEANYYSNATTDEHGLVQFSINTTNVMGTSLTVRVNYKDRSPCYGYQWVSEEHEEAHHTAYLVFSPSKSFVHLEPMSHELPCGHTQTVQAHYILNGGTLLGLKKLSFYYLIMAKGGIVRTGTHGLLVKQEDMKGHFSISIPVKSDIAPVARLLIYAVLPTGDVIGDSAKYDVENCLANKVDLSFSPSQSLPASHAHLRVTAAPQSVCALRAVDQSVLLMKPDAELSASSVYNLLPEKDLTGFPGPLNDQDDEDCINRHNVYINGITYTPVSSTNEKDMYSFLEDMGLKAFTNSKIRKPKMCPQLQQYEMHGPEGLRVGFYESDVMGRGHARLVHVEEPHTETVRKYFPETWIWDLVVVNSAGVAEVGVTVPDTITEWKAGAFCLSEDAGLGISSTASLRAFQPFFVELTMPYSVIRGEAFTLKATVLNYLPKCIRVSVQLEASPAFLAVPVEKEQAPHCICANGRQTVSWAVTPKSLGNVNFTVSAEALESQELCGTEVPSVPEHGRKDTVIKPLLVEPEGLEKETTFNSLLCPSGGEVSEELSLKLPPNVVEESARASVSVLGDILGSAMQNTQNLLQMPYGCGEQNMVLFAPNIYVLDYLNETQQLTPEVKSKAIGYLNTGYQRQLNYKHYDGSYSTFGERYGRNQGNTWLTAFVLKTFAQARAYIFIDEAHITQALIWLSQRQKDNGCFRSSGSLLNNAIKGGVEDEVTLSAYITIALLEIPLTVTHPVVRNALFCLESAWKTAQEGDHGSHVYTKALLAYAFALAGNQDKRKEVLKSLNEEAVKKDNSVHWERPQKPKAPVGHFYEPQAPSAEVEMTSYVLLAYLTAQPAPTSEDLTSATNIVKWITKQQNAQGGFSSTQDTVVALHALSKYGAATFTRTGKAAQVTIQSSGTFSSKFQVDNNNRLLLQQVSLPELPGEYSMKVTGEGCVYLQTSLKYNILPEKEEFPFALGVQTLPQTCDEPKAHTSFQISLSVSYTGSRSASNMAIVDVKMVSGFIPLKPTVKMLERSNHVSRTEVSSNHVLIYLDKVSNQTLSLFFTVLQDVPVRDLKPAIVKVYDYYETDEFAIAEYNAPCSKDLGNA*

>A2M L606K M607D

MGKNKLLHPSLVLLLLVLLPTDASVSGKPQYMVLVPSLLHTETTEKGCVLLSYLNETVTVSASLESVRGNRSLFTDLEAENDVLHCVAFAVPKSSSNEEVMFLTVQVKGPTQEFKKRTTVMVKNEDSLVFVQTDKSIYKPGQTVKFRVVSMDENFHPLNELIPLVYIQDPKGNRIAQWQSFQLEGGLKQFSFPLSSEPFQGSYKVVVQKKSGGRTEHPFTVEEFVLPKFEVQVTVPKIITILEEEMNVSVCGLYTYGKPVPGHVTVSICRKYSDASDCHGEDSQAFCEKFSGQLNSHGCFYQQVKTKVFQLKRKEYEMKLHTEAQIQEEGTVVELTGRQSSEITRTITKLSFVKVDSHFRQGIPFFGQVRLVDGKGVPIPNKVIFIRGNEANYYSNATTDEHGLVQFSINTTNVMGTSLTVRVNYKDRSPCYGYQWVSEEHEEAHHTAYLVFSPSKSFVHLEPMSHELPCGHTQTVQAHYILNGGTLLGLKKLSFYYLIMAKGGIVRTGTHGLLVKQEDMKGHFSISIPVKSDIAPVARLLIYAVLPTGDVIGDSAKYDVENCLANKVDLSFSPSQSLPASHAHLRVTAAPQSVCALRAVDQSVLKDKPDAELSASSVYNLLPEKDLTGFPGPLNDQDDEDCINRHNVYINGITYTPVSSTNEKDMYSFLEDMGLKAFTNSKIRKPKMCPQLQQYEMHGPEGLRVGFYESDVMGRGHARLVHVEEPHTETVRKYFPETWIWDLVVVNSAGVAEVGVTVPDTITEWKAGAFCLSEDAGLGISSTASLRAFQPFFVELTMPYSVIRGEAFTLKATVLNYLPKCIRVSVQLEASPAFLAVPVEKEQAPHCICANGRQTVSWAVTPKSLGNVNFTVSAEALESQELCGTEVPSVPEHGRKDTVIKPLLVEPEGLEKETTFNSLLCPSGGEVSEELSLKLPPNVVEESARASVSVLGDILGSAMQNTQNLLQMPYGCGEQNMVLFAPNIYVLDYLNETQQLTPEVKSKAIGYLNTGYQRQLNYKHYDGSYSTFGERYGRNQGNTWLTAFVLKTFAQARAYIFIDEAHITQALIWLSQRQKDNGCFRSSGSLLNNAIKGGVEDEVTLSAYITIALLEIPLTVTHPVVRNALFCLESAWKTAQEGDHGSHVYTKALLAYAFALAGNQDKRKEVLKSLNEEAVKKDNSVHWERPQKPKAPVGHFYEPQAPSAEVEMTSYVLLAYLTAQPAPTSEDLTSATNIVKWITKQQNAQGGFSSTQDTVVALHALSKYGAATFTRTGKAAQVTIQSSGTFSSKFQVDNNNRLLLQQVSLPELPGEYSMKVTGEGCVYLQTSLKYNILPEKEEFPFALGVQTLPQTCDEPKAHTSFQISLSVSYTGSRSASNMAIVDVKMVSGFIPLKPTVKMLERSNHVSRTEVSSNHVLIYLDKVSNQTLSLFFTVLQDVPVRDLKPAIVKVYDYYETDEFAIAEYNAPCSKDLGNA*
